# Examining the Influence of Nondimensionalization on Partial Rank Correlation Coefficient Results when Modeling the Epithelial Mesenchymal Transition

**DOI:** 10.1007/s11538-024-01393-y

**Published:** 2024-12-19

**Authors:** Kelsey I. Gasior

**Affiliations:** Department of Applied and Computational Mathematics and Statistics, University of Notre Dame, Notre Dame, IN USA

**Keywords:** EMT, Sensitivity analysis, Nondimensionalization, Latin hypercube sampling, Partial rank correlation coefficient

## Abstract

Partial Rank Correlation Coefficient (PRCC) is a powerful type of global sensitivity analysis. Usually performed following Latin Hypercube Sampling (LHS), this analysis can highlight the parameters in a mathematical model producing the observed results, a crucial step when using models to understand real-world phenomena and guide future experiments. Recently, Gasior et al. performed LHS and PRCC when modeling the influence of cell–cell contact and TGF-$\beta$ signaling on the epithelial mesenchymal transition (Gasior et al. in J Theor Biol 546:111160, 2022). Though their analysis provided insight into how these tumor-level factors can impact intracellular signaling during the transition, their results were potentially impacted by nondimensionalizing the model prior to performing sensitivity analysis. This work seeks to understand the true impact of nondimensionalization on sensitivity analysis by performing LHS and PRCC on both the original model that Gasior et al. proposed and seven different nondimensionalizations. Parameter ranges were kept small to capture shifts in the values that originally produced bistable behavior. By comparing these eight different iterations, this work shows that the issues from performing sensitivity analysis following nondimensionalization are two-fold: (1) nondimensionalization can obscure or exclude important parameters from in-depth analysis and (2) how a model is nondimensionalized can, potentially, change analysis results. Ultimately, this work cautions against using nondimensionalization prior to sensitivity analysis if the subsequent results are meant to guide future experiments.

## Introduction

Sensitivity analysis is a powerful tool to determine the source of model uncertainty by exploring the complicated dynamics underlying the model and quantifying the impact of the parameters on observed outputs  (Saltelli et al. 2008). As models describing real world phenomena help guide future experiments, this process can highlight whether parameters in a model are uncertain due to gaps in collected data or inherent variations in the structure of the model  (Gasior and Cogan 2024; Smith 2013). There are two main types of sensitivity analysis: local and global analysis. Local analysis requires that one parameter is varied at a time while global analysis allows for varying all parameters simultaneously  (Jarrett et al. 2015; Campolongo et al. 2011). Thus global sensitivity analysis examines how changes in the model output can be the result of one parameter, or a combination of parameters, but it can also be computationally expensive  (Campolongo et al. 2011; Qian and Mahdi 2020). Further, the results of any sensitivity analysis are impacted by the techniques performed, the parameter ranges explored, and the parameter distributions  (Qian and Mahdi 2020).

One type of global sensitivity analysis method is Partial Rank Correlation Coefficient (PRCC), which can be performed following parameter sampling techniques, such as Latin Hypercube Sampling (LHS)  (Qian and Mahdi 2020; Blower and Dowlatabadi 1994). LHS is a type of Monte Carlo Sampling where each parameter is randomly sampled from its own probability distribution function. The marginal distribution of each parameter is sampled *N* times. Each value of a parameter is only used once and the values are assembled into *N* different model input vectors to produce the necessary *N* outputs, at which time PRCC is performed  (Blower and Dowlatabadi 1994; McKay et al. 1979). In nonlinear models, for each input parameter and output, PRCC ranks the value and quantifies the monotonic behavior of the data  (Qian and Mahdi 2020; Gomero 2012). PRCC requires a monotonic relationship between the individual input parameters and the model output  (Blower and Dowlatabadi 1994). If monotonic behavior with respect to each parameter does not occur, the results may be unreliable  (Qian and Mahdi 2020; Blower and Dowlatabadi 1994). Thus, in a nonlinear model, fulfilling these conditions could be time intensive when performing PRCC.

Recent work by Gasior et al.  (2022) employed LHS and PRCC to understand a mathematical model of the epithelial mesenchymal transition (EMT) in MCF7 breast carcinoma cells. EMT is a process by which epithelial cells undergo phenotypic and behavioral changes to acquire the invasive and migratory properties associated with mesenchymal cells  (Nieto et al. 2016; Kalluri and Weinberg 2009; Kalluri 2009). In response to cues from the surrounding microenvironment, intracellular signaling pathways are upregulated and shift the cell from its normally adhesive nature to the mesenchymal phenotype, allowing it to migrate away from the tumor and, potentially, form a metastasis  (Gonzalez and Medici 2014). It has been hypothesized that there is a bistable switch underlying EMT, allowing a transitioned cell to maintain the mesenchymal phenotype in the absence of the original signal  Gasior et al. (2022), Gasior et al. (2017). In their work, Gasior et al. explored the roles of the TGF-$\beta$ signaling pathway and cell–cell contact on EMT via an ordinary differential equation (ODE) model of E-cadherin and Slug, two important intracellular components  (Gasior et al. 2022). E-cadherin is a pro-epithelial factor associated with cellular adhesion in epithelial cells and Slug is a pro-mesenchymal transcription factor downstream of the TGF-$\beta$ pathway  (Christofori and Semb 1999; Taylor et al. 2010). The authors hypothesized that there were two bistable switches that would allow for an epithelial cell to transition; a reversible switch with respect to the loss of cell–cell contact and an irreversible switch from exposure to exogenous TGF-$\beta$. They showed the paths through which cells with varying cell–cell contact can undergo EMT when exposed to TGF-$\beta$ and then analyzed their model using LHS and PRCC. Through their analysis, the authors found that loss of cellular contact and increased exposure to exogenous TGF-$\beta$ shifted the dependence of E-cadherin and Slug from rates controlling their own natural production and degradation rates to the degradation rate of Slug. This work highlighted the mechanisms underlying the bistable switch in these breast carcinoma cells and the importance of these natural rates of production and degradation rates, depending on the stage of cell transition  (Gasior et al. 2022).

While the model put forth by Gasior et al. provided insight into the impact of cell–cell contact and TGF-$\beta$ signaling on intracellular factors, their analysis was potentially affected by nondimensionalizing their model prior to carrying out LHS and PRCC  (Gasior et al. 2022). Nondimensionalization is a mathematical technique that uses substitution and reduction to define a model solely in terms of nondimensional variables and parameters that represent new parameter groupings the original model  (Conesa et al. 2016). Depending on the system, this technique can allow for more accurate analysis  (Pérez et al. 2017). Due to the unknown values of their intracellular rates, Gasior et al. performed nondimensionalization and reduced their parameter set to six nondimensional parameters prior to their analysis  (Gasior et al. 2022). While this technique helped determine the behavior underlying the system, it potentially obscured and reduced the efficacy of the sensitivity analysis results. Additionally, defining these dimensional constants is a choice made by the authors and produced groupings for the nondimensional parameters that are specific to the final model presented. This choice could, again, cast doubt on the impact of certain parameters on the observed dynamics.

This work revisits the sensitivity analysis performed by Gasior et al. by examining the impact of nondimensionalization on PRCC analysis. Rather than nondimensionalize the model with one specific set of choices for the parameters, this work will perform LHS and PRCC on both the original, dimensional model proposed by Gasior et al. and on seven different nondimensionalized systems. Similar to the technique Gasior et al. performed on their nondimensionalized system, this work uses uniform distributions for LHS on all dimensionalized and nondimensionalized parameters. Additionally, while Gasior et al. explored wide parameter ranges for LHS to encompass the entire bistable region, this analysis examines a small range ($\pm 10\%$ of the value) around the parameter value that resulted in the bistable switch. This methodical analysis reveals that, for cells lacking the ability to transition with respect to TGF-$\beta$ due to their level of cellular contact, the choice of nondimensionalization does not matter, as all variations of the model produce the same results. Rather, it is in systems where bistable behavior occurs that choices in nondimensionalization can impact the nondimensional parameters deemed important to the level of E-cadherin. Further, by nondimensionalizing the model, Gasior et al. missed parameters that could impact the steady state values of E-cadherin and Slug at key positions in the tumor. The loss of knowledge on these parameters could lead to missteps in future EMT experiments. Ultimately, this work highlights that nondimensionalization should not be carried out prior to performing PRCC and that caution must be applied when obtaining information on nondimensional groups of parameters.

## Methods

### Model of E-Cadherin & Slug During EMT

**Fig. 1:**
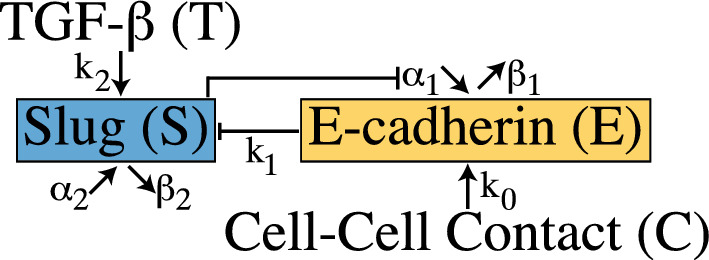
In the presence of cellular contact ($$C>0$$, $$T = 0 $$), E-cadherin moves to the membrane, forming intracellular bonds with $$\beta $$-catenin and interacting with E-cadherin molecules on neighboring cells. These interactions prevent $$\beta $$-catenin from translocating to the nucleus, keeping Slug, a transcription factor, at low concentrations in epithelial cells. Exposure to exogenous TGF-$$\beta $$ ($$T>0$$) triggers an intracellular signaling cascade that activates Slug, which then suppresses E-cadherin production. The loss of E-cadherin at the membrane allows $$\beta $$-catenin to translocate to the nucleus and further activate Slug

Gasior et al. put forth a two equation ODE model of membrane-bound E-cadherin (*E*), and the transcription factor Slug (*S*) found in Equations 1–2  (Gasior et al. 2022). A schematic of this model is shown in Fig. 1 and includes production and degradation of both E-cadherin ($\alpha _1$, $\beta _1$, respectively) and Slug ($\alpha _2$, $\beta _2$, respectively). In the presence of other cells, E-cadherin migrates to the cell membrane to form adhesion complexes and adhere the cell to its neighbors by interacting with E-cadherin molecules on nearby cells  (Christofori and Semb 1999; Ramis-Conde et al. 2008). Without the presence of this cell–cell contact, E-cadherin is endocytosed  (Ramis-Conde et al. 2008). The movement of E-cadherin to the membrane is modeled by $\displaystyle \frac{k_0 \big (\frac{C}{IC_C}\big )^{n_2}}{1+\big (\frac{C}{IC_C}\big )^{n_2}}$, where *C* is cell–cell contact and is a continuous input parameter. Once at the membrane, E-cadherin binds with members of the catenin family, such as $\beta$-catenin, to link it to the actin cytoskeleton  (Christofori and Semb 1999; Ramis-Conde et al. 2008; Semb and Christofori 1998). These cadherin-catenin interactions sequester $\beta$-catenin at the membrane and, with the constant degradation of free $\beta$-catenin by the GSK-3$\beta$ complex, it is unable to move to the nucleus, thus suppressing Slug  (Ramis-Conde et al. 2008; Heuberger and Birchmeier 2010). This suppression is modeled by $\displaystyle \frac{k_1 \big (\frac{E}{IC_E}\big )^{n_3}}{1+\big (\frac{E}{IC_E}\big )^{n_3}}$.

Upon the release of TGF-$\beta$ (*T*) from the surrounding microenvironment, the ligand activates an intracellular signaling cascade that targets Slug  (Taylor et al. 2010; Naber et al. 2013; Parvani et al. 2011). Slug activation via the TGF-$\beta$ signaling pathway is modeled by $\displaystyle \frac{k_2 \big (\frac{T}{I_T}\big )^{n_4}}{1+\big (\frac{T}{I_T}\big )^{n_4}}$. Activation of Slug then inhibits E-cadherin transcription  Conacci-Sorrell et al. (2003), which is modeled via $\displaystyle \frac{\alpha _1}{1+\big (\frac{S}{IC_S}\big )^{n_1}}$. All parameter values are shown in Table 1. Note, some parameter values are slightly different from the work published in Gasior et al, as the parameter values originally presented by the authors were rounded. The model was run in MATLAB and XPPAUT. Initial conditions for all 8 treatment groups explored can be found in Table 5. Code available upon request.

1 $$\begin{aligned} \frac{dE}{dt}&= \frac{\alpha _1}{1+\big (\frac{S}{IC_S}\big )^{n_1}} + \frac{k_0 \big (\frac{C}{IC_C}\big )^{n_2}}{1+\big (\frac{C}{IC_C}\big )^{n_2}}-\beta _1E \end{aligned}$$

2 $$\begin{aligned} \frac{dS}{dt}&= \alpha _2 -\frac{k_1 \big (\frac{E}{IC_E}\big )^{n_3}}{1+\big (\frac{E}{IC_E}\big )^{n_3}}+\frac{k_2 \big (\frac{T}{IC_T}\big )^{n_4}}{1+\big (\frac{T}{IC_T}\big )^{n_4}}-\beta _2S \end{aligned}$$

**Fig. 2:**
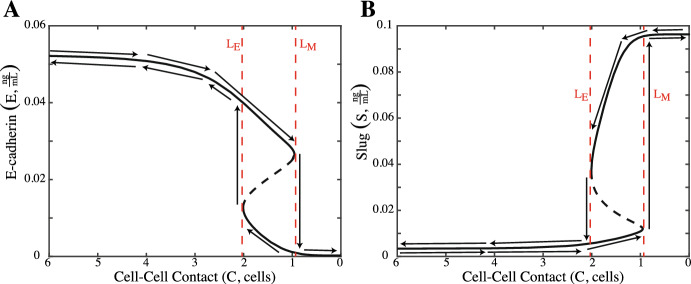
**A**, **B** The reversible bistable switch of E-cadherin (*E*) and Slug (*S*) with respect to a loss of cell–cell contact when $T=0 \frac{{{\text {ng}}}}{{{\text {mL}}}}$. A cell with 6 neighbors ($C=6$ cells) begins in the epithelial steady state with a high concentration of membrane-bound E-cadherin ($E=0.0522 \frac{{{\text {ng}}}}{{{\text {mL}}}}$) and a low concentration of Slug ($S=0.0034 \frac{{{\text {ng}}}}{{{\text {mL}}}}$). Loss of cell–cell contact reduces the concentration of membrane-bound E-cadherin and increases the presence of Slug. Once a sufficient amount of cellular contact has been lost ($C=0.9532$ cells), as indicated by $L_M$, the cell transitions to the mesenchymal state. If the cell can regain enough cellular contact ($C = 2.0090$ cells), it can transition back to the epithelial phenotype ($L_E$)

Figure 2 shows the bifurcation analysis with respect to loss of cell–cell contact (*C*) for E-cadherin and Slug in cells without exposure to TGF-$\beta$ (). Here, cells with 6 neighbors begin in the epithelial steady state. Loss of neighbors and cellular contact decreases the amount of membrane-bound E-cadherin and increases the amount of Slug until the cell transitions to the mesenchymal phenotype at $L_M$ when $C=0.9532$ cells. But, if the cell were to regain neighbors, it could transition back to the epithelial state at $L_E$ when $C=2.0090$ cells. Thus, with respect to cellular contact, the bistable switch between the epithelial and mesenchymal steady states is reversible.

**Table 1:** Parameter Definitions for E-cadherin-Slug ODE Model

Parameter	Definition	Assumed value	Range	Units	Source
$\alpha _1$	E-cadherin production	0.19355	[0.1742, 0.2129]	$\frac{{{\text {ng}}}}{{{\text {mL}} \cdot {\text {min}}}}$	Estimated
$\alpha _2$	Slug production	0.080	[0.0772, 0.0932]	${\frac{{{\text {ng}}}}{{{\text {mL}}} \cdot {\text {min}}}}$	Estimated
$\beta _1$	E-cadherin degradation	6.035	[5.4315, 6.6385]	$\frac{1}{\text {min}}$	Estimated
$\beta _2$	Slug degradation	0.830	[0.7470, 0.9130]	$\frac{1}{\text {min}}$	Estimated
$k_0$	Rate at which E-cadherin moves to the membrane due to cell–cell contact	0.1245	[0.1120, 0.1370]	$\frac{{{\text {ng}}}}{{{\text {mL}}} \cdot {\text {min}}}$	Estimated
$k_1$	Rate of Slug suppression from membrane-bound E-cadherin activation	0.080	[0.0669, 0.0829]	$\frac{{{\text {ng}}}}{{{\text {mL}}} \cdot {\text {min}}}$	Estimated
$k_2$	Rate of Slug upreglation via TGF-$\beta$	0.040	[0.0360, 0.0440]	$\frac{{{\text {ng}}}}{{{\text {mL}} \cdot {\text {min}}}}$	Estimated
$IC_S$	Half maximal concentration of Slug required to inhibit E-cadherin production	0.01920	[0.0173, 0.0211]	$\frac{{{\text {ng}}}}{{{\text {mL}}}}$	Estimated
$IC_E$	Half maximal concentration of E-cadherin required to inhibit Slug upregulation	0.010	[0.0090, 0.0110]	$\frac{{{\text {ng}}}}{{{\text {mL}}}}$	Estimated
$IC_T$	Half maximal concentration of TGF-$\beta$ required to activate Slug	$3.64 \times 10^{-6}$	$[3.2760 \times 10^{{ - 6}} ,\,4.0040 \times 10^{{ - 6}} ]$	$\frac{{{\text {ng}}}}{{{\text {mL}}}}$	Estimated
$IC_C$	Half maximal number of cells required to draw E-cadherin to the membrane for activation	2.17	[1.9530, 2.3870]	cells	Estimated
$n_1$	Hill coefficient	3	–	–	Estimated
$n_2$	Hill coefficient	4	–	–	Estimated
$n_3$	Hill coefficient	2	–	–	Estimated
$n_4$	Hill coefficient	3	–	–	Estimated

**Fig. 3:**
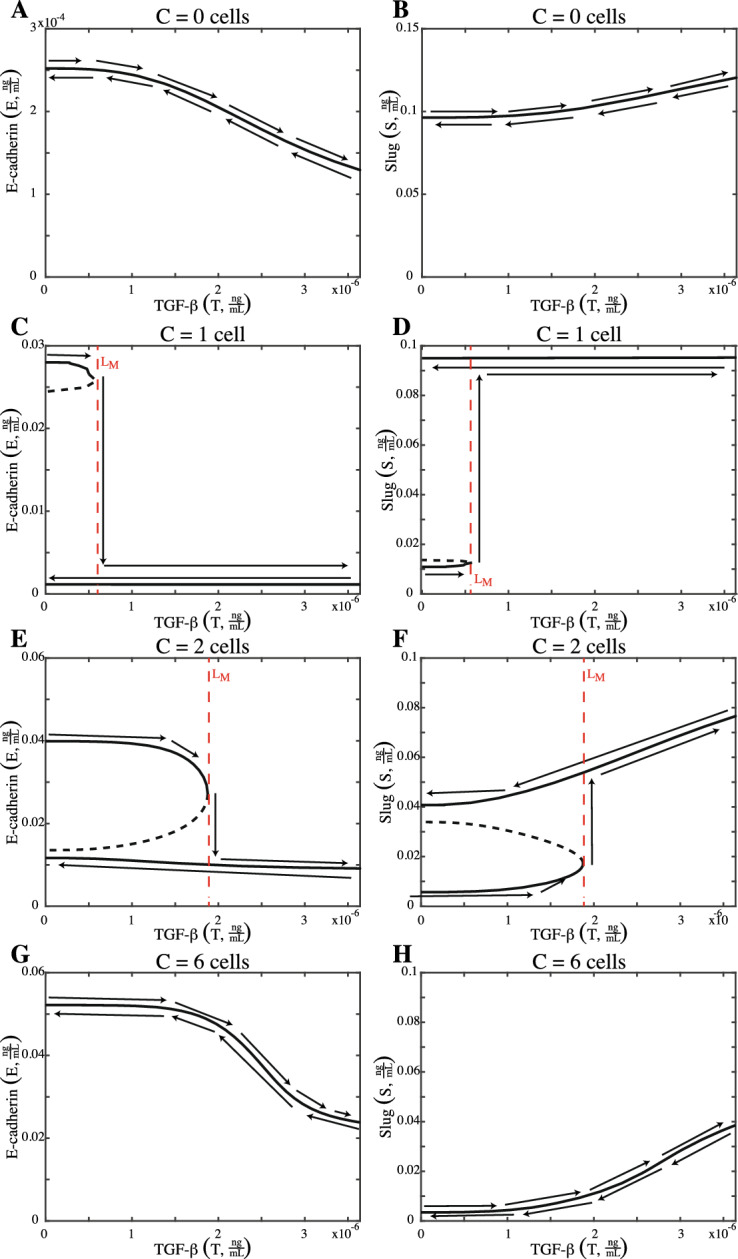
**A**, **B** Cells without cellular contact ($C=0$ cells) begin in the mesenchymal state with low membrane E-cadherin ($E \approx 0.0003 \frac{{{\text {ng}}}}{{{\text {mL}}}}$) and a high Slug ($S \approx 0.0963 \frac{{{\text {ng}}}}{{{\text {mL}}}}$). Exogenous TGF-$\beta$ (*T*) lowers *E* and increases *S* but does not produce a switch. **C**, **D** Cells with 1 neighbor ($C=1$ cell) begin in the epithelial state. Increasing TGF-$\beta$ reduces E-cadherin and increases Slug. Once a threshold of $T \approx 5.0224 \times 10 ^{-7} \frac{{{\text {ng}}}}{{{\text {mL}}}}$ is reached ($(E,S)\approx (0.0266,0.0121) \frac{{{\text {ng}}}}{{{\text {mL}}}}$), the cell transitions to the mesenchymal steady state and is unable to transition back to the epithelial state if TGF-$\beta$ is reduced, making it an irreversible switch. **E**, **F** Epithelial cells with 2 neighbors ($C=2$ cells) must receive $T \approx 1.8757 \times 10^{-6} \frac{{{\text {ng}}}}{{{\text {mL}}}}$ ($(E,S)\approx (0.0273, 0.0172) \frac{{{\text {ng}}}}{{{\text {mL}}}}$) for the cell to undergo the irreversible switch to the mesenchymal state. **G**, **H** Epithelial cells with 6 neighbors ($C=6$ cells) are unable to transition as TGF-$\beta$ is increased

Bistability due to changes in exogenous TGF-$\beta$ is shown in Fig. 3. In Fig. 3A, B, cells without cell–cell contact ($C=0$ cells) begin in the mesenchymal phenotype and then maintain it after exposure to TGF-$\beta$. Cells with one (Fig. 3C, D) or two neighbors (Fig. 3E, F) now begin in the epithelial phenotype and exposure to TGF-$\beta$ results in an irreversible switch to the mesenchymal phenotype. Once these cells obtain the mesenchymal steady state, they cannot transition back to the epithelial state when exogenous TGF-$\beta$ is lost. For cells with one neighbor (Fig. 3C, D), this switch requires $T \approx 5.0224 \times 10 ^{-7} \frac{{{\text {ng}}}}{{{\text {mL}}}}$. For cells with two neighbors, there is a higher threshold of $T \approx 1.8757 \times 10^{-6} \frac{{{\text {ng}}}}{{{\text {mL}}}}$ (Fig. 3E, F). When cell–cell contact is increased to $C=6$ cells, the cell will lose some membrane-bound E-cadherin and have an increase in Slug when exposed to TGF-$\beta$, but will not undergo the bistable switch.

### Nondimensionalization

Due to the theoretical nature of this model and the estimated parameter values (Table 1), Gasior et al. nondimensionalized Equations 1–2  (Gasior et al. 2022). The variables E-cadherin (*E*), Slug (*S*), and time (*t*), as well as the input parameters of cellular contact (*C*) and exogenous TGF-$\beta$ (*T*) are defined in terms of a dimensional parameter ($\epsilon$, $\sigma$, $\gamma$, $\phi$, and $\pi$, respectively) and a nondimensional variable (*e*, *s*, $\tau$, $\mu$, and $\theta$, respectively) as shown in Eqs. 3.

3 $$\begin{aligned} E = \epsilon \cdot e, \quad \quad S=\sigma \cdot s, \quad \quad t = \gamma \tau , \quad \quad C = \phi \cdot \mu , \quad \quad T = \pi \cdot \theta \end{aligned}$$

Gasior et al. chose the half-maximal values for E-cadherin ($IC_E$), Slug ($IC_S$), cellular contact ($IC_C$), and TGF-$\beta$ ($IC_T$) for the definitions of *E*, *S*, *C*, *T*, respectively:

4 $$\begin{aligned} E = IC_E \cdot e, \quad \quad S=IC_S \cdot s, \quad \quad C = IC_C \cdot \mu , \quad \quad T = IC_T \cdot \theta \end{aligned}$$

Thus, the system became:

5 $$\begin{aligned} \frac{de}{d\tau }&= \bigg (\frac{\alpha _1\gamma }{I_E}\bigg )\bigg (\frac{1}{1+s^{n_1}}\bigg ) + \bigg (\frac{k_0\gamma }{I_E}\bigg )\bigg (\frac{\mu ^{n_2}}{1+\mu ^{n_2}}\bigg )-\beta _1\gamma e \end{aligned}$$

6 $$\begin{aligned} \frac{ds}{d\tau }&= \bigg (\frac{\alpha _2\gamma }{I_S}\bigg ) -\bigg ( \frac{k_1\gamma }{I_S}\bigg )\bigg (\frac{e^{n_3}}{1+e^{n_3}}\bigg ) + \bigg ( \frac{k_2\gamma }{I_S}\bigg )\bigg (\frac{\theta ^{n_4}}{1+\theta ^{n_4}}\bigg ) -\beta _2\gamma s \end{aligned}$$

Now, there are seven different possible parameter combinations for $\gamma$. These options are presented in Table 2. Any choice for $\gamma$ would result in the nondimensional model:

7 $$\begin{aligned} \frac{de}{d\tau }&= \frac{A_1}{1+s^{n_1}} + K_0 \bigg (\frac{\mu ^{n_2}}{1+\mu ^{n_2}}\bigg )-B_1 e \end{aligned}$$

8 $$\begin{aligned} \frac{ds}{d\tau }&= A_2 -K_1 \bigg (\frac{e^{n_3}}{1+e^{n_3}}\bigg ) + K_2 \bigg (\frac{\theta ^{n_4}}{1+\theta ^{n_4}}\bigg ) -B_2 s \end{aligned}$$

Depending on the choice of $\gamma$, the nondimensional parameters in Eqs. 7-8 would have different meanings and values, as shown in Table 3. Any $\gamma$ parameter combination from Table 2 still produces a reversible bistable switch with respect to cell–cell contact ($\mu$) and an irreversible switch with respect to TGF-$\beta$ ($\theta$), as shown in Appendix Figs. 6, 7, 8, 9, 10, 11 and 12.

**Table 2:** Possible choices for $\gamma$

	$\gamma$	Definition
Set 1	$\frac{I_E}{\alpha _1}$	Ratio of half maximal concentration of E-cadherin required to inhibit Slug upregulation to E-cadherin production
Set 2	$\frac{I_E}{k_0}$	Ratio of half maximal concentration of E-cadherin required to inhibit Slug upregulation to the rate at which E-cadherin moves to the membrane due to cell–cell contact
Set 3	$\frac{1}{\beta _1}$	Inverse of E-cadherin degradation rate
Set 4	$\frac{I_S}{\alpha _2}$	Ratio of half maximal concentration of Slug required to inhibit E-cadherin production to Slug production
Set 5	$\frac{I_S}{k_1}$	Ratio of half maximal concentration of Slug required to inhibit E-cadherin production to the rate of Slug suppression from membrane-bound E-cadherin activation
Set 6	$\frac{I_S}{k_2}$	Ratio of half maximal concentration of Slug required to inhibit E-cadherin production to the rate of Slug upregulation via TGF-$\beta$
Set 7	$\frac{1}{\beta _2}$	Inverse of Slug degradation rate

**Table 3:** Nondimensional parameter definitions and values

		${A_1}$	${K_0}$	${B_1}$	${A_2}$	${K_1}$	${K_2}$	${B_2}$
Set 1	Definition		$\frac{k_0}{\alpha _1}$	$\frac{\beta _1IC_E}{\alpha _1}$	$\frac{\alpha _2 IC_E}{\alpha _1IC_S}$	$\frac{k_1IC_E}{\alpha _1IC_S}$	$\frac{k_2IC_E}{\alpha _1IC_S}$	$\frac{\beta _2IC_E}{\alpha _1}$
	Value	**1**.**00**	0.646	0.312	0.215	0.215	0.108	0.043
Set 2	Definition	$\frac{\alpha _1}{k_0}$		$\frac{\beta _1IC_E}{k_0}$	$\frac{\alpha _2IC_E}{k_0IC_S}$	$\frac{k_1IC_E}{k_0IC_S}$	$\frac{k_2IC_E}{k_0IC_S}$	$\frac{\beta _2IC_E}{k_0}$
	Value	1.555	**1**.**00**	0.485	0.335	0.335	0.167	0.067
Set 3	Definition	$\frac{\alpha _1}{\beta _1 IC_E}$	$\frac{k_0}{\beta _1 IC_E}$		$\frac{\alpha _2}{\beta _1IC_S}$	$\frac{k_1}{\beta _1IC_S}$	$\frac{k_2}{\beta _1IC_S}$	$\frac{\beta _2}{\beta _1}$
	Value	3.207	2.063	**1**.**00**	0.690	0.690	0.345	0.138
Set 4	Definition	$\frac{\alpha _1IC_S}{\alpha _2IC_E}$	$\frac{k_0IC_S}{\alpha _2IC_E}$	$\frac{\beta _1IC_S}{\alpha _2}$		$\frac{k_1}{\alpha _2}$	$\frac{k_2}{\alpha _2}$	$\frac{\beta _2IC_S}{\alpha _2}$
	Value	4.645	2.988	1.448	**1**.**00**	1.000	0.500	0.199
Set 5	Definition	$\frac{\alpha _1IC_S}{k_1IC_E}$	$\frac{k_0IC_S}{k_1IC_E}$	$\frac{\beta _1IC_S}{k_1}$	$\frac{\alpha _2}{k_1}$		$\frac{k_2}{k_1}$	$\frac{\beta _2IC_S}{k_1}$
	Value	4.645	2.988	1.448	1.000	**1**.**00**	0.500	0.199
Set 6	Definition	$\frac{\alpha _1IC_S}{k_2IC_E}$	$\frac{k_0IC_S}{k_2IC_E}$	$\frac{\beta _1IC_S}{k_2}$	$\frac{\alpha _2}{k_2}$	$\frac{k_1}{k_2}$		$\frac{\beta _2IC_S}{k_2}$
	Value	9.290	5.976	2.897	2.000	2.000	**1**.**00**	0.398
Set 7	Definition	$\frac{\alpha _1}{\beta _2IC_E}$	$\frac{k_0}{\beta _2IC_E}$	$\frac{\beta _1}{\beta _2}$	$\frac{\alpha _2}{\beta _2IC_S}$	$\frac{k_1}{\beta _2IC_S}$	$\frac{k_2}{\beta _2IC_S}$	
	Value	23.319	15.000	7.271	5.020	5.020	2.510	**1**.**00**

### Latin Hypercube Sampling and Partial Rank Correlation Coefficient in Dimensional Model

Latin Hypercube Sampling and Partial Rank Correlation Coefficient were used to determine the sensitivity of E-cadherin (*E*) and Slug (*S*) steady state values on the 11 parameters in Eqs. 1, 2. Each parameter was varied over a range of $\pm 10\%$ of the parameter value and these ranges are also in Table 1. LHS requires that the *K* parameter spaces must be sampled *N* times with the condition that $N > \frac{4}{3}K$  (Blower and Dowlatabadi 1994). For a model with 11 varied parameters, $N > 15$.

The model was exposed to two levels of TGF-$\beta$ ($T = 0 \frac{{{\text {ng}}}}{{{\text {mL}}}}$, $T = 3.64 \times 10^{-6} \frac{{{\text {ng}}}}{{{\text {mL}}}})$ and four levels of cell–cell contact ($C = 0$, 1, 2, and 6 cells) for eight total treatment groups. To adequately sample the parameter space, $N=10000$ samples were chosen from uniform parameter distributions and randomly assembled into parameter sets. For each level of cell–cell contact, across both levels of TGF-$\beta$, initial conditions were established corresponding to the model in Eqs. 1, refeq2 and are listed in Table 5. Time course simulations were run in MATLAB. E-cadherin (*E*) and Slug (*S*) values were then recorded at $t = 10000$ min and rounded to the nearest $10^{-4}$ to ensure monotonic behavior. PRCC then transforms input parameter samples and the E-cadherin and Slug steady states into ranked values and measures the correlation between the rank transformed input parameters and rank-transformed outcomes. PRCC between the 11 parameters for all treatment groups revealed $|\rho _{ij}|<0.5$ $\forall$ $i \ne j = 1, \ldots , 11$, indicating that there are not hidden relationships between the parameters of Eqs. 1-2. All code available upon request.

Note that PRCC requires that each output produce monotonic behavior when each parameter is varied individually  (Blower and Dowlatabadi 1994). The monotonicity plots for all 11 parameters are shown in the Appendix Figs. 13, 14, 15, 16. For the model in Eqs. 1, 2, the parameter ranges for $\alpha _2$ and $k_1$ in Table 1 have been shifted from the original range of $\pm 10\%$ of the value. While the overall range size for both parameters is maintained, they are no longer centered about the original value due to a lack of monotonicity and negative E-cadherin and Slug values when varied individually.

**Table 4:** Nondimensional parameter ranges

	${A_1}$	${K_0}$	${B_1}$	${A_2}$	${K_1}$	${K_2}$	${B_2}$
Set 1	**X**	[0.5814, 0.7106]	[0.2808, 0.3432]	[0.2073, 0.2503]	[0.1800, 0.2230]	[0.0972, 0.1188]	[0.0387, 0.0473]
Set 2	[1.3995, 1.7105]	**X**	[0.4365, 0.5335]	[0.3230, 0.3900]	[0.2805, 0.3475]	[0.1503, 0.1837]	[0.0603, 0.0737]
Set 3	[2.8863, 3.5277]	[1.8567, 2.2693]	**X**	[0.6651, 0.8031]	[0.5778, 0.7158]	[0.3105, 0.3795]	[0.1242, 0.1518]
Set 4	[4.1805, 5.1095]	[2.6892, 3.2868]	[1.3032, 1.5928]	**X**	[0.8364, 1.0364]	[0.4500, 0.5500]	[0.1791, 0.2189]
Set 5	[4.1805, 5.1095]	[2.6892, 3.2868]	[1.3032, 1.5928]	[0.9649, 1.1649]	**X**	[0.4500, 0.5500]	[0.1791, 0.2189]
Set 6	[8.3610, 10.2190]	[5.3784, 6.5736]	[2.6073, 3.1867]	[1.9277, 2.3277]	[1.6750, 2.0750]	**X**	[0.3582, 0.4378]
Set 7	[20.9871, 25.6509]	[13.5000, 16.5000]	[6.5439, 7.9981]	[4.8384, 5.8424]	[4.2043, 5.2083]	[2.2590, 2.7610]	**X**

An X indicates that the parameter was not varied and was held constant at a value of 1.00 for the set

### Latin Hypercube Sampling and Partial Rank Correlation Coefficient in Nondimensional Models

Latin Hypercube Sampling and Partial Rank Correlation Coefficient were used to determine the sensitivity of nondimensional E-cadherin (*e*) and Slug (*s*) steady state values on the seven different nondimensional models put forth by Table 2 and Eqs. 7-8. Each parameter was varied over a range of $\pm 10\%$ of the values in Table 3 and these ranges are in Table 4. A parameter range of $\text {X}$ in Table 4 indicates that this parameter was held constant at a value of 1.00 for this set. For Sets 1–7, $K=6$ and, thus, $N > 8$.

The models were exposed to two levels of nondimensional TGF-$\beta$ ($\theta = 0$, $\theta = 1$) and four levels of nondimensional cell–cell contact ($\mu = 0$, 0.46, 0.92, 2.76) for eight total treatment groups per model. To adequately sample the parameter space, $N=10000$ samples were chosen from uniform parameter distributions and randomly assembled into parameter sets. For each level of cell–cell contact, across both levels of TGF-$\beta$, initial conditions were established corresponding to the model in Eqs. 7-8 and are listed in Table 5. Time course simulations were run in MATLAB. E-cadherin (*e*) and Slug (*s*) values were then recorded at $\tau = 10000$ and rounded to the nearest $10^{-2}$ to ensure monotonic behavior. PRCC then transforms input parameter samples and the E-cadherin (*e*) and Slug (*s*) steady states into ranked values and measures the correlation between the rank transformed input parameters and rank-transformed outcomes. PRCC in Sets 1–7 for all treatment groups revealed $|\rho _{ij}|<0.5$ $\forall$ $i \ne j = 1, \ldots , 6$, indicating that there are not hidden relationships between the parameters of Eqs. 7-8 for any nondimensionalization. All code available upon request.

The monotonicity plots for Sets 1–7 models are shown in the Appendix Figs. 17, 18, 19, 20, 21, 22, 23, 24, 25, 26, 27, 28, 29, 30. For all models, the parameter ranges for $A_2$ and $K_1$ have been shifted from the original range of $\pm 10\%$ of the value in Table 3, as reflected in Table 4. While the overall range size for all shifted parameters is maintained, they are no longer centered about the original value due to a lack of monotonicity and negative E-cadherin and Slug values when varied individually.

**Table 5:** Initial Conditions for PRCC analysis

Dimensional model			Sets 1–7		
	*E*(0) ($\frac{{{\text {ng}}}}{{{\text {mL}}}}$)	*S*(0) ($\frac{{{\text {ng}}}}{{{\text {mL}}}}$)		*e*(0)	*s*(0)
C = 0 cells	0.0003	0.0963	$\mu = 0$	0.0251	5.0220
C = 1 cell	0.0280	0.0109	$\mu = 0.46$	2.7923	0.5712
C = 2 cells	0.0399	0.0057	$\mu = 0.92$	3.9869	0.2974
C = 6 cell	0.0522	0.0034	$\mu = 2.76$	5.2185	0.1780

**Fig. 4:**
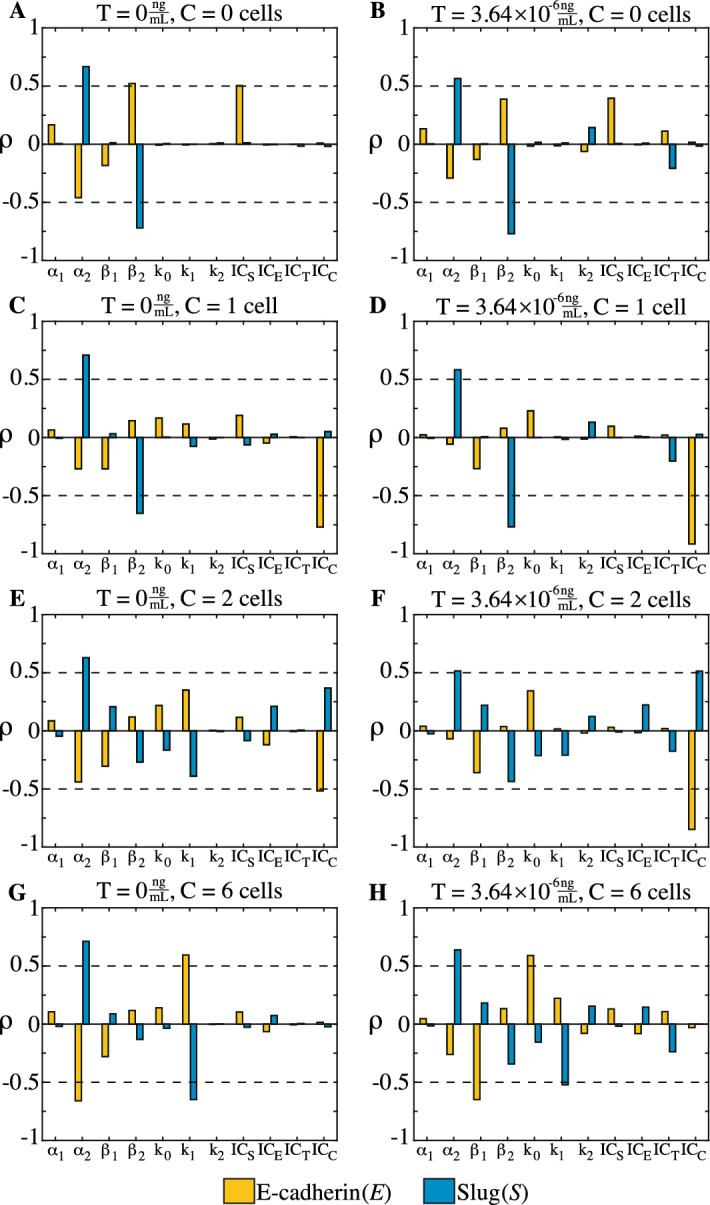
PRCC results of the 11 parameters from Eqs. 1, 2 on the steady state values of E-cadherin (*E*) and Slug (*S*) for 2 levels of TGF-$\beta$ ($T=0$, $3.64 \times 10^{-6} \frac{{{\text {ng}}}}{{{\text {mL}}}}$) and four levels of cellular contact ($C=0$, 1, 2, and 6 cells). E-cadherin is in gold and Slug is in blue. Cut off for significance is marked in dashed lines for each treatment group ($|\rho | = 0.5$). For all treatment groups, Slug is dependent on its own production rate. The dependence of Slug on the level of cellular contact (*C*) is clear, as increasing *C* shifts the importance from the degradation rate of Slug ($\beta _2$) to the suppression of Slug by E-cadherin at the membrane ($k_1$). The parameters impacting E-cadherin depend on both the level of cellular contact and the amount of exogenous TGF-$\beta$ present. As both TGF-$\beta$ and cellular contact are increased, the steady state value of E-cadherin shifts from depending on Slug-related parameters (A) to E-cadherin-related parameters (H). (color figure online)

## Results

### PRCC for Dimensional Model Highlights the Importance of Exogenous Factors on E-Cadherin and Slug Steady State Values

Figure 4 shows the PRCC results for the impact of the 11 parameters from Eqs. 1, 2 on the steady state values of E-cadherin (*E*) and Slug (*S*). Values of $\rho = -0.5$ and $\rho = 0.5$ are used as cutoffs when determining significant parameter impact on E-cadherin and Slug steady state values. For Slug (*S*), Fig. 4 highlights a shift as the number of neighbors is increased from $C=0$ cells to $C=6$ cells. When $C=0$ cells, the parameters influencing the steady state value of Slug (*S*) are the rate of Slug production ($\alpha _2$) and degradation ($\beta _2$). While the dependence of Slug on $\alpha _2$ remains constant for all treatment groups, increasing the number of neighbors results in the importance of degradation ($\beta _2$) receding. Instead, the rate at which Slug is suppressed by membrane-bound E-cadherin ($k_1$) begins to significantly impact the value of Slug. This behavior is maintained for both values of TGF-$\beta$, indicating that it is the movement of E-cadherin to the membrane that is suppresing the value of Slug by preventing $\beta$-catenin from activating Slug in these highly epithelial cells. Thus, cell–cell contact is another underlying factor affecting Slug.

Changes in parameter influence on the steady state value of E-cadherin (*E*) depend on both the value of TGF-$\beta$ and cell–cell contact. In the absence of TGF-$\beta$ ($T=0 \frac{{{\text {ng}}}}{{{\text {mL}}}}$), as shown in Fig. 4, the degradation of Slug ($\beta _2$) and the half maximal concentration of Slug necessary to inhibit E-cadherin production ($IC_S$) are the parameters impacting the steady state value of E-cadherin (*E*). Increasing the number of neighbors then pushes the cell into the epithelial phenotype, changing parameter influence. For $C=1-2$ cells, without TGF-$\beta$, dependence on Slug related parameters recedes and, instead, the only parameter that significantly affects E-cadherin is the half maximal number of cells required to draw E-cadherin to the membrane for activation ($IC_C$). With this increase in cellular contact, the cell moves from depending on Slug-related parameters to a parameter that can determine whether a cell exists in the epithelial steady state. As the cellular neighbor number is increased further ($C=6$ cells), E-cadherin depends on the production rate of Slug ($\alpha _2$) and the rate of Slug suppression from membrane-bound E-cadherin activation ($k_1$) in the absence of TGF-$\beta$ ($T = 0\frac{{{\text {ng}}}}{{{\text {mL}}}}$). Thus, the cell has renewed dependence on Slug-associated parameters, as changes to the production of Slug ($\alpha _2$) or its suppression ($k_1$) could alter Slug-accumulation and subsequent E-cadherin levels prior to the addition of TGF-$\beta$.

Exposing the cell to exogenous TGF-$\beta$ results in a dependence of E-cadherin on E-cadherin-related parameters as cell–cell contact is increased. For cells without cellular contact ($C=0$ cells), the addition of TGF-$\beta$ ($T=3.64 \times 10^{-6} \frac{{{\text {ng}}}}{{{\text {mL}}}}$) reveals that none of the parameters surpass the threshold necessary to significantly influence E-cadherin. For $C=1-2$ cells, just like cells without TGF-$\beta$, the only parameter that significantly impacts E-cadherin is the half maximal number of cells required to draw E-cadherin to the membrane for activation ($IC_C$). When contact is increased to $C=6$ cells, E-cadherin levels depend on their own degradation rate ($\beta _1$) and the rate at which E-cadherin is activated by moving to the membrane due to cell–cell contact ($k_0$). Thus, in the presence of TGF-$\beta$ ($T=3.64 \times 10^{-6} \frac{{{\text {ng}}}}{{{\text {mL}}}}$) increasing the number of neighbors highlights the importance of parameters associated with the presence of E-cadherin at the membrane on the steady state E-cadherin value.

**Fig. 5:**
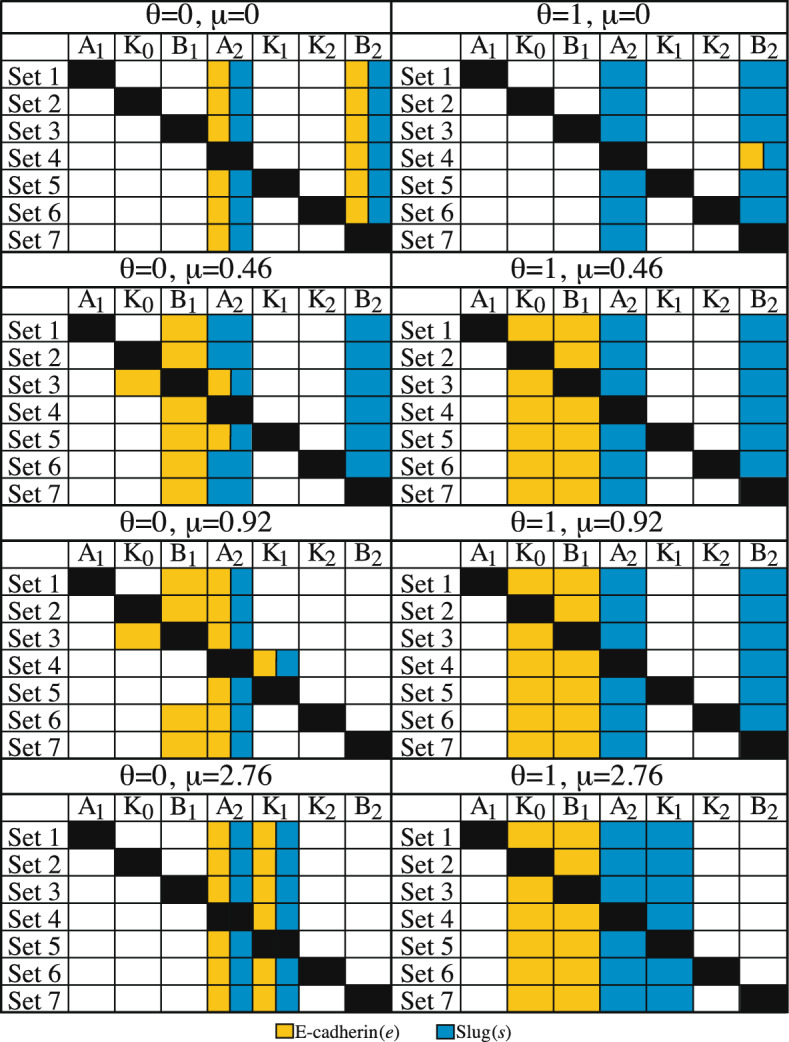
Summary of PRCC results for nondimensionalized Sets 1–7 for parameter impact on the steady state value of nondimensional E-cadherin (*e*) and Slug (*s*) under two levels of TGF-$\beta$ ($\theta =0$, 1) and four levels of cellular contact ($\mu =0$, 0.46, 0.92, 2.76). Boxes marked with black indicate that the parameter was not varied. Full PRCC results can be found in Appendix Figs. 31, 32, 33, 34, 35, 36, 37. A gold box indicates that the impact of the parameter on *e* exceeds $|\rho |=0.5$ and a blue box if the impact on *s* exceeds $|\rho |=0.5$. This figure highlights the dependence of Slug on cellular contact ($\mu$), as increasing $\mu$ shifts the importance from the production and degradation rates of Slug ($A_2$, $B_2$) to production and the suppression of Slug by E-cadherin at the membrane ($A_2$, $K_1$). The parameters impacting the steady state value of nondimensional E-cadherin depend both on the cellular conditions and, for some treatment groups ($\theta =0$; $\mu =0.46$, 0.92), the choice in nondimensionalization.(color figure online)

### PRCC for Nondimensionalized Systems Shows that Exogenous Factors and Nondimensionalization Choices Impact Intracellular Steady State Values

A summary of the PRCC results for nondimensionalized Sets 1–7 is shown in Fig. 5. Here, depending on the nondimensional value chosen for $\gamma$, the six parameter definitions vary (as shown in Table 3) but their positions within the model do not. These results examine the impact of each parameter on the steady state value of nondimensional E-cadherin (*e*) and Slug (*s*) when exposed to nondimensional exogenous TGF-$\beta$ ($\theta = 0$, 1) and nondimensional cell–cell contact ($\mu =0$, 0.46, 0.92, 2.76). Boxes marked with black indicate that the parameter was not varied, as it was the group of parameters that time *t* was nondimensionalized with and corresponds to a value of 1.00 in Table 4. Values of $|\rho | =0.5$ are used as cutoffs when determining important parameters for *e* and *s*. A gold box indicates that the impact of the parameter on *e* exceeds these thresholds. A blue box indicates that the impact of the parameter on the steady state of *s* exceeds these thresholds. The full results of PRCC on all nondimensionalizations can be found in Appendix Figs. 31, 32, 33, 34, 35, 36, 37.

For the nondimensional steady state value of Slug (*s*), PRCC of nondimensional Sets 1–7 highlights a similar dependence on cellular contact ($\mu$) as occurred in the dimensional system. As shown in Fig. 5, for all treatment groups and all nondimensionalizations, Slug is impacted by its own nondimensional production ($A_2$). For cells without cell–cell contact ($\mu = 0$), the steady state of Slug is also dependent upon its nondimensional degradation rate ($B_2$). However, increasing cellular contact shifts the dependence from the nondimensional degradation of Slug ($B_2$) to the nondimensional rate at which Slug is suppressed by E-cadherin due to membrane activation ($K_1$). This behavioral pattern occur at both levels of TGF-$\beta$ ($\theta$) and is consistent through all seven nondimensionalizations, indicating that it is, potentially, the original parameters, $\alpha _2$, $\beta _2$, and $k_1$ that are controlling the behavior of Slug as cell–cell contact changes.

The nondimensional E-cadherin (*e*) steady state is more susceptible to changes in cell–cell contact ($\mu$) and TGF-$\beta$ ($\theta$). For cells without exogenous TGF-$\beta$, changes in cell–cell contact shift parameter importance but maintain a dependence on Slug-related activity. As shown in Fig. 5, for both cells without any neighbors (($\mu$, $\theta )=(0,$ 0)), the two most important parameters controlling *e* are the nondimensional rates of production ($A_2$) and degradation ($B_2$) for Slug (*s*). Increasing $\mu$ to simulate a highly epithelial cell with 6 neighbors ($\mu =2.76$) maintained the impact of the production rate of Slug ($A_2$) on E-cadherin but shifted from Slug degradation to the rate at which E-cadherin can suppress Slug due to its activation at the membrane ($K_1$), a result similar to what was observed in the dimensional model.

Adding TGF-$\beta$ to the system changes the most influential parameters on E-cadherin from Slug-associated rates to E-cadherin-associated parameters. With the addition of exogenous TGF-$\beta$ ($\mu = 0$, $\theta = 1$), Fig. 5 shows that, for almost all sets of nondimensionalization, there is not a parameter that significantly impacts the steady state values of nondimensional E-cadherin (*e*). But, for cells with at least one cellular neighbor ($\mu \ge 0.46$) and the addition of TGF-$\beta$ ($\theta =1$), the steady state value of nondimensional E-cadherin (*e*) does not fluctuate with the way the system is nondimensionalized, nor with added cell–cell contact. Here, the *e* depends on the rate of E-cadherin degradation ($B_1$) and the rate at which E-cadherin moves to the membrane due to cell–cell contact ($K_0$). The lack of change due to different nondimensionalizations indicates that it is, potentially, the original dimensional parameters $\beta _1$ and $k_0$, respectively, that are controlling the steady-state value.

Unlike Slug, PRCC analysis for E-cadherin is also susceptible to variations in nondimensionalization. For cells with 1 neighbor and lacking exogenous TGF-$\beta$ (($\mu$, $\theta )=(0.46,$ 0)), one important parameter controlling the steady state value (*e*) is $B_1$ for all sets where it is varied. Whether a second influential parameter is identified depends on the nondimensionalization. In Set 3, $B_1$ is held constant and PRCC highlights the importance of $K_0$ and $A_2$ on *e*. Note that the definition of $K_0$ in Set 3 is the inverse of the Set 2 definition of $B_1$. For Set 5, both $B_1$ and $A_2$ are influencing the level of *e*. One possible explanation is that, in Set 5, the definition of $A_2$ is the ratio of $\alpha _2$ to $k_1$, which are the two parameter ranges in the dimensional model that were shifted due to non-monotonic behavior and negative values.

Increasing cellular contact to $(\mu ,\theta )=(0.92,0)$ also presents variations in PRCC results based on nondimensionalization. For all sets where it is varied, one important parameter controlling E-cadherin (*e*) is the nondimensional production rate for Slug ($A_2$). Similar to systems with (($\mu$, $\theta )=(0.46,$ 0), whether a second parameter is identified by PRCC is determined by the nondimensionalization. For Sets 1,2,6,7 the most important parameters controlling *e* are $B_1$ and $A_2$. However, different results arise for Sets 3–5. In Set 3, $B_1$ is held constant and, now, $K_0$ is now important to the value of *e*. Here, the definition of $K_0$ in Set 3 is the inverse of the definition of $B_1$ in Set 2. Set 4 and 5 hold $A_2$ and $K_1$ constant, respectively, while other parameters are varied, with $K_1=\frac{k_1}{\alpha _2}$ in Set 4 and $A_2 = \frac{\alpha _2}{k_1}$ in Set 5. These are the two parameter ranges that were adjusted. Thus, depending on the nondimensionalization, three potential factors can influence and change the results of PRCC for the model.

## Discussion

This work reexamines the sensitivity analysis performed by Gasior et al. on a nondimensionalized model of the intracellular dynamics governing EMT in response to activation of the TGF-$\beta$ signaling pathway and loss of cell–cell contact. In their original work, Gasior et al. nondimensionalized their model of E-cadherin-Slug dynamics and then performed LHS and PRCC on the resulting model  (Gasior et al. 2022). While this work resulted in a better understanding of the E-cadherin- and Slug-associated rates that govern their steady state values under varying levels of cellular contact and TGF-$\beta$, their methodology missed key results. Here, LHS and PRCC is applied directly to the original model to determine the impact of 11 dimensional parameters. Additionally, the model was nondimensionalized in seven different ways to determine whether a specific iteration of nondimensionalization would have preserved the analysis results from Eqs. 1-2. This methodology reveals that preceding sensitivity analysis with nondimensionalization could alter the results of PRCC and, further, choices in nondimensionalization could also alter the observations made and conclusions drawn.

One difference between performing PRCC on the dimensional model and the nondimensionalized models (Sets 1–7) was the loss of understanding on which parameters are impacting the steady state values of E-cadherin and Slug under different tumor conditions. There are commonalities in behavior between the nondimensional models and the original model, such as the lack of sensitive parameters impacting E-cadherin when the cell lacks neighbors ($C=0$ cells, $\mu =0$) and exogenous TGF-$\beta$ added ($T=3.64 \times 10^{-6} \frac{{{\text {ng}}}}{{{\text {mL}}}}$, $\theta = 1$), as well as the overall shift in dependence of Slug from its own degradation rate ($\beta _2$, $B_2$) to the rate at which E-cadherin suppress Slug due to its movement to the membrane ($k_1$, $K_1$) as cellular contact is increased. Further, the model from Eqs. 1-2 and nondimensionalized Sets 1–7 captured the impact of cell–cell contact on Slug and both contact and TGF-$\beta$ on E-cadherin. However, nondimensionalizing the model using all four half-maximal inhibitory concentrations (*IC*) for the dimensional parameter in E-cadherin ($IC_E$), Slug ($IC_S$), cellular contact ($IC_C$), and TGF-$\beta$ ($IC_T$) means that the sensitivity analysis cannot determine the importance of these parameters. Specifically, $IC_C$ and $IC_T$ do not appear in any of the parameter combinations for any of the nondimensionalizations because cellular contact and TGF-$\beta$ are input parameters. Thus, while the half maximal number of cells necessary for E-cadherin activation at the membrane is the only parameter impacting E-cadherin for $C=1-2$ cells, the influence of this parameter is completely lost in nondimensionalization. Instead, PRCC performed on nondimensionalized systems highlights other parameters. For example, in nondimensionalized systems with $\mu =0.46$, 0.92 and with exogenous TGF-$\beta$ ($\theta = 1$), PRCC indicates that the nondimensional degradation rate of E-cadherin ($B_1$) and the rate at which E-cadherin is activated at the membrane due to cell–cell contact ($K_0$) are impacting the steady state value of E-cadherin (*e*). However, when comparing these results to the dimensional model, it is clear that neither $\beta _1$ nor $k_0$ reach the threshold of $|\rho | \ge 0.5$, leading to a false understanding of which parameters control the value of E-cadherin in these two treatment groups. Indeed, this loss of knowledge goes even further, as Fig. 4 shows Slug depends on $IC_C$ in cells with 2 cellular neighbors ($C=2$ cells) and exogenous TGF-$\beta$ applied ($T=3.64 \times 10^{-6} \frac{{{\text {ng}}}}{{{\text {mL}}}}$).

The grouping of certain parameters together also means that the individual influence of each parameter is lost with nondimensionalization. Figure 4 shows that, for cells without any neighbors ($C=0$ cells) and without exogenous TGF-$\beta$ ($T=0 \frac{{{\text {ng}}}}{{{\text {mL}}}}$), the steady state of E-cadherin (*E*) is sensitive to the degradation rate of Slug ($\beta _2$) and the half maximal amount of Slug necessary to inhibit E-cadherin transcription ($IC_S$). However, when the system is nondimensionalized, that same treatment group ($\mu =0$, $\theta =0$) highlights the importance of $A_2$, the nondimensional rate of Slug production in Sets 1–7. Thus, nondimensionalization could lead to the conclusion that it is the production rate of Slug ($\alpha _2$) that is the most important parameter impacting E-cadherin in the treatment group when, in actuality, the dimensional system shows that this parameter does not reach the significance threshold of $|\rho | \ge 0.5$.

In addition to missing influential parameters, choices in sampling and nondimensionalization can affect the results. In this case study, a uniform distribution was used for LHS sampling for all nondimensional parameters to (1) test the methodology put forth by Gasior et al. and (2) simulate the common choices that could be made when applying LHS and PRCC. However, given that the dimensional model parameters in Eqs. 1-2 were uniformly distributed, it would potentially be more appropriate to use different parameter distributions for the nondimensional parameters. Nondimensional parameter groupings combine multiple uniform distributions, producing parameters with nonuniform distributions  Kinderman and Monahan (1977), Sakamoto (1943). These different distributions could potentially produce varying results in sensitivity. Specifically, these nonuniform distributions could potentially cause issues in cases where cells had 1–2 neighbors ($\mu =0.46$, 0.92). Here, the cell initially exists in the epithelial steady state region where bistable behavior is present ($\theta =0$). Two-parameter bifurcation diagrams for the nondimensionalized system in Gasior et al. shows that the value chosen is close to the boundary of the bistable region. The $\pm 10\%$ parameter value range that was used captures parameter values in both the bistable region and the monostable region. For this work, Fig. 5 shows that nondimensionalization choices, even with a uniform parameter distribution, can change the results for E-cadherin (*e*) in these treatment groups. A skewed parameter distribution that over- or under-samples the bistable region could, potentially effect these results further. Thus, preceding PRCC with nondimensionalization in regions where important behavioral changes occur could be problematic and unreliable as it (1) depends on the modeling choices made and (2) excludes parameters from analysis. The potential errors introduced via uninformed analysis could then subsequently produce errors when it is used to inform wet lab experiments.

Ultimately, this work shows that performance of LHS and PRCC cannot be preceded by nondimensionalization of a mathematical model. While nondimensionalization can help with understanding the overall behavior of the system, as well as numerical issues that can arise during analysis, it fails to allow for substantive and correct understanding of the dynamics underlying the real world phenomena. While PRCC on a nondimensionalized system can show how parameter impact changes with respect to different treatment groups, its accuracy is unreliable once the model is in this form, especially for systems that model bistable behavior. While one potential cause for the discrepancies could be the parameter range around the original value, and future work should explore the importance of this parameter range in the context of the biological system and the subsequently formed analysis, in biomathematical models where parameters are difficult to measure, analyzing the original system with dimensional parameters is the best course of action.

## Figures and Tables

**Fig. 6:**
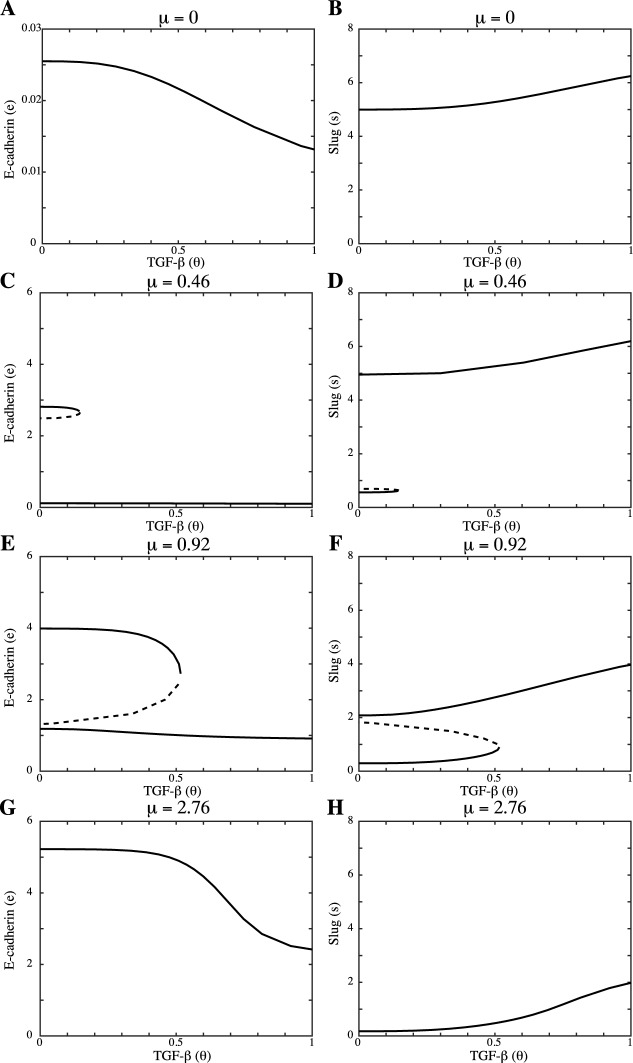
Bifurcation plots for E-cadherin (*e*) and Slug (*s*) with respect to $\theta$ at (**A**, **B**) $\mu =0$, (**C**, **D**) $\mu =0.46$, (**E**, **F**) $\mu =0.92$, and (**G**, **H**) $\mu =2.76$ for nondimensionalized Set 1

**Fig. 7:**
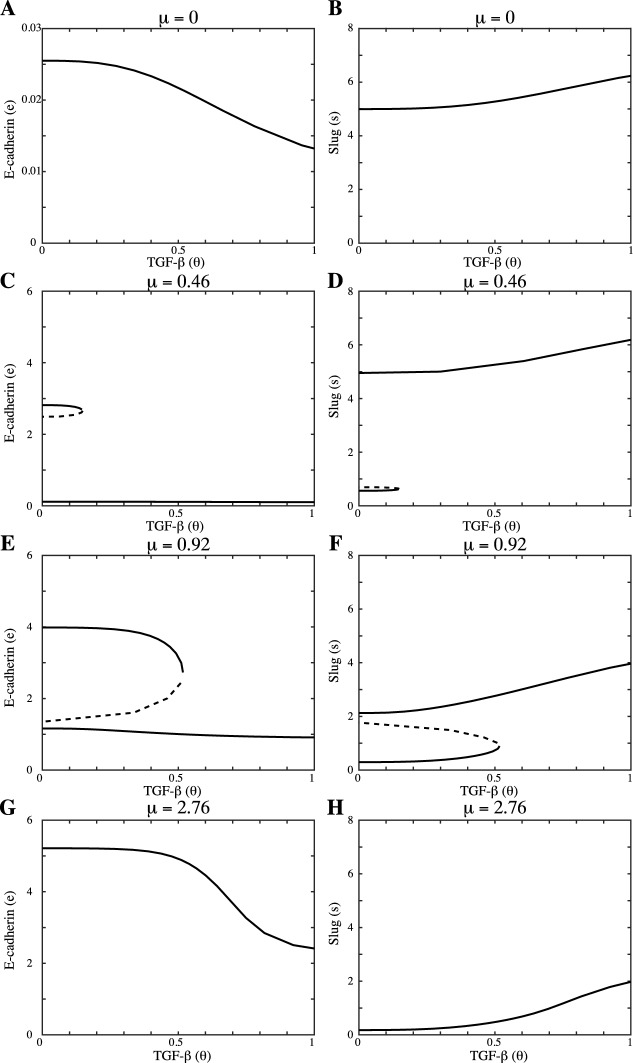
Bifurcation plots for E-cadherin (*e*) and Slug (*s*) with respect to $\theta$ at (**A**, **B**) $\mu =0$, (**C**, **D**) $\mu =0.46$, (**E**, **F**) $\mu =0.92$, and (**G**, **H**) $\mu =2.76$ for nondimensionalized Set 2

**Fig. 8:**
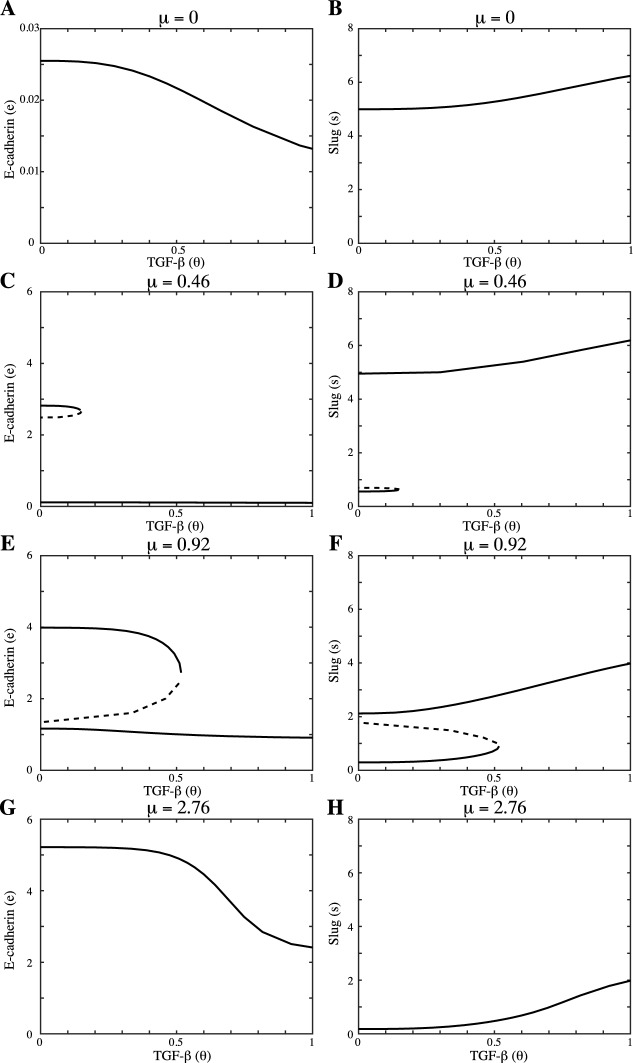
Bifurcation plots for E-cadherin (*e*) and Slug (*s*) with respect to $\theta$ at (**A**, **B**) $\mu =0$, (**C**, **D**) $\mu =0.46$, (**E**, **F**) $\mu =0.92$, and (**G**, **H**) $\mu =2.76$ for nondimensionalized Set 3

**Fig. 9:**
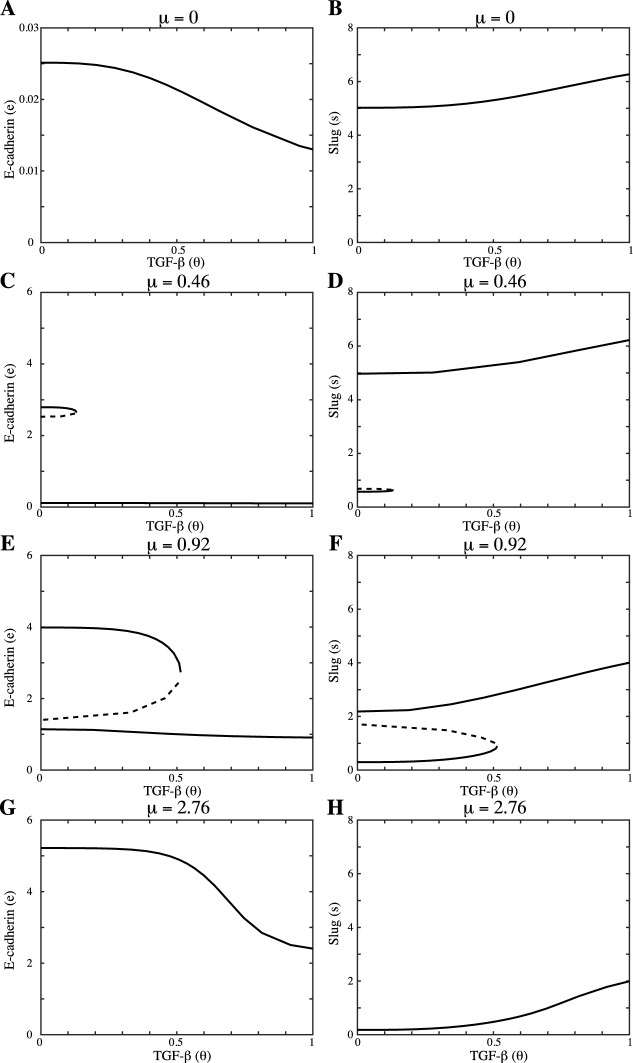
Bifurcation plots for E-cadherin (*e*) and Slug (*s*) with respect to $\theta$ at (**A**, **B**) $\mu =0$, (**C**, **D**) $\mu =0.46$, (**E**, **F**) $\mu =0.92$, and (**G**, **H**) $\mu =2.76$ for nondimensionalized Sets 4 and 5

**Fig. 10:**
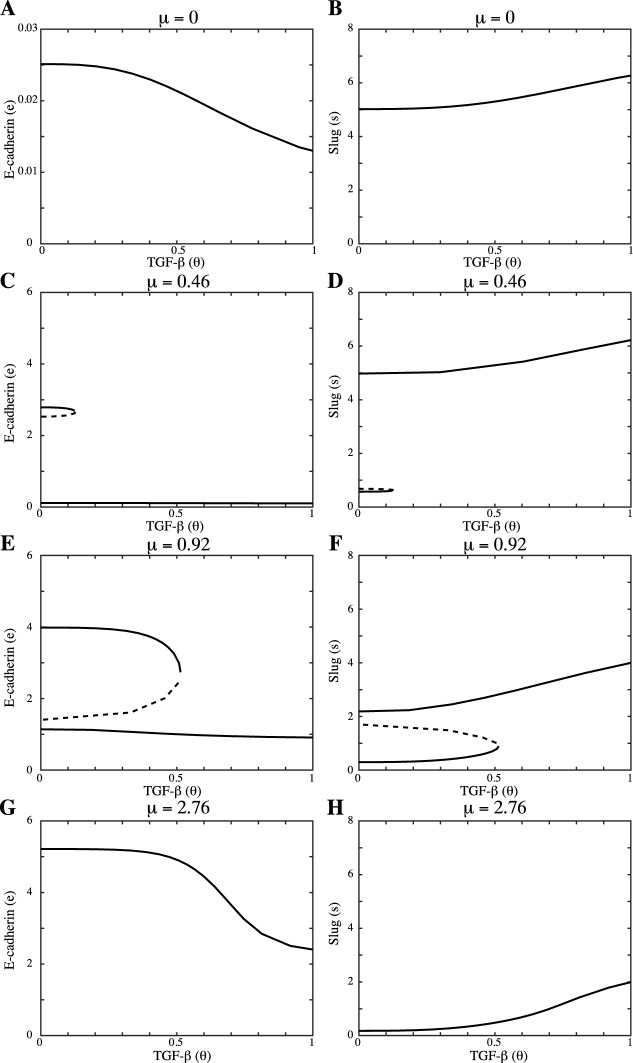
Bifurcation plots for E-cadherin (*e*) and Slug (*s*) with respect to $\theta$ at (**A**, **B**) $\mu =0$, (**C**, **D**) $\mu =0.46$, (**E**, **F**) $\mu =0.92$, and (**G**, **H**) $\mu =2.76$ for nondimensionalized Set 6

**Fig. 11:**
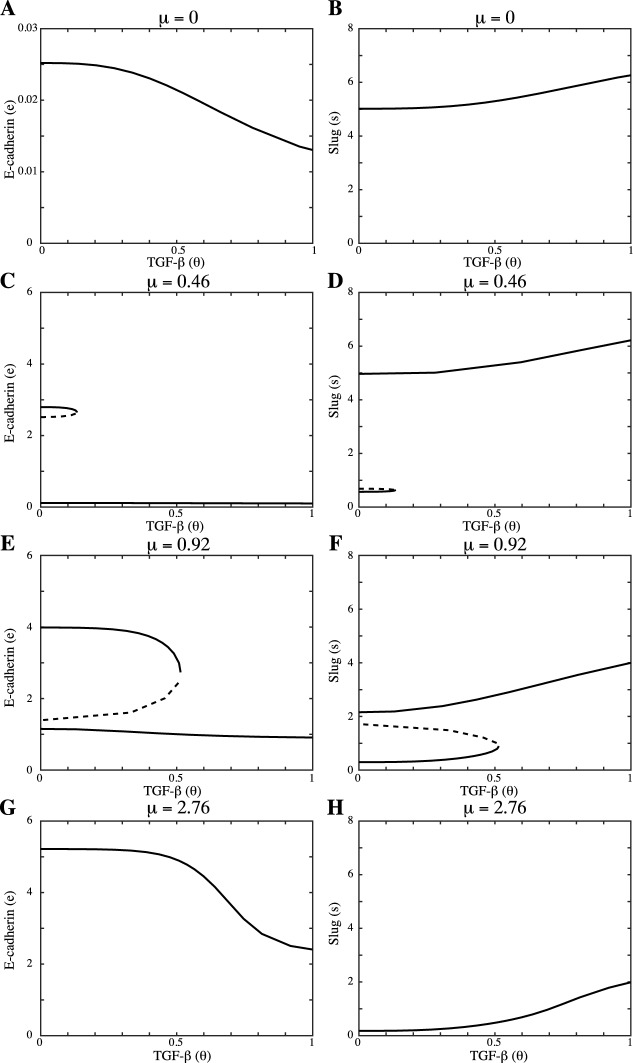
Bifurcation plots for E-cadherin (*e*) and Slug (*s*) with respect to $\theta$ at (**A**, **B**) $\mu =0$, (**C**, **D**) $\mu =0.46$, (**E**, **F**) $\mu =0.92$, and (**G**, **H**) $\mu =2.76$ for nondimensionalized Set 7

**Fig. 12:**
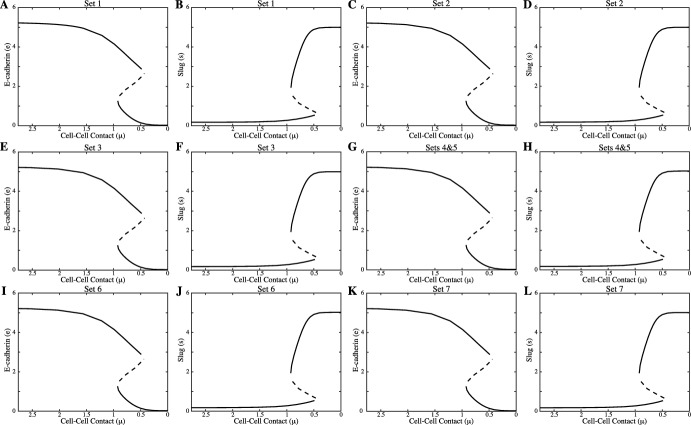
Bifurcation plots for E-cadherin (*e*) and Slug (*s*) with respect to $\mu$ with $\theta =0$ for nondimensionalized Sets 1–7

**Fig. 13:**
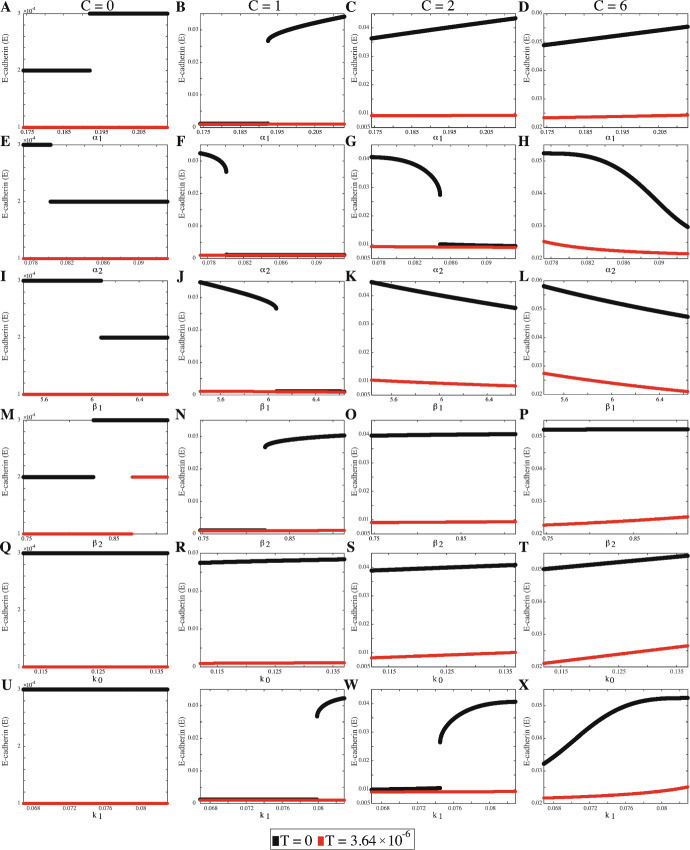
E-cadherin (*E*) monotonicity plots for parameters (**A**–**D**) $$\alpha _1$$, (**E**–**H**) $$\alpha _2$$, (**I**–**L**) $$\beta _1$$, (**M**–**P**) $$\beta _2$$, (**Q**–**T**) $$k_0$$, and (**U**–**X**) $$k_1$$ from Eqs. 1, 2. For each plot, $$T=0 $$ is in black and and $$T=3.64 \times 10^{-6} $$ is in red. The level of cell–cell contact is given for each column with (**A**, **E**, **I**, **M**, **Q**, **U**) $$C=0$$ cells, (**B**, **F**, **J**, **N**, **R**, **V**) $$C=1$$ cells, (**C**, **G**, **K**, **O**, **S**, **W**) $$C=2$$ cells, and (**D**, **H**, **L**, **P**, **T**, **X**) $$C=6$$ cells (color figure online)

**Fig. 14:**
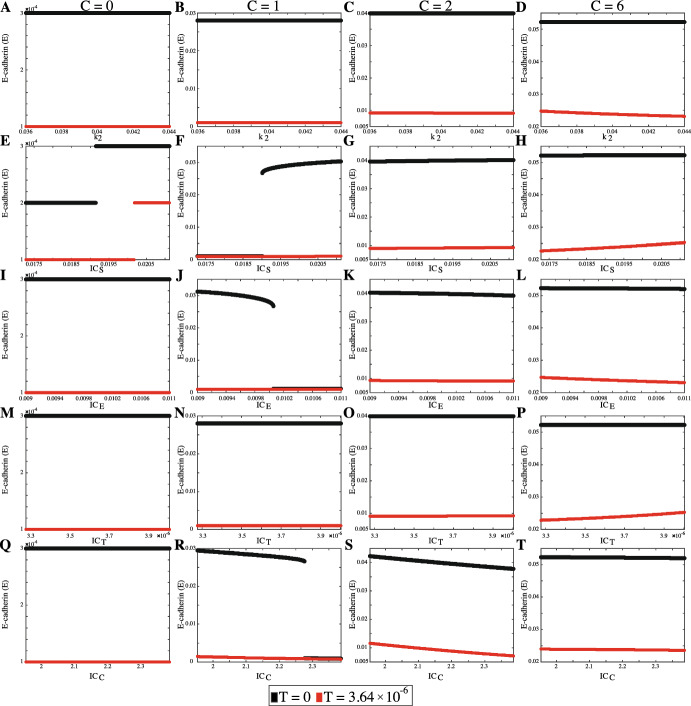
E-cadherin (*E*) monotonicity plots for parameters (**A**–**D**) $$k_2$$, (**E**–**H**) $$IC_S$$, (**I**–**L**) $$IC_E$$, (**M**–**P**) $$IC_T$$, and (**Q**–**T**) $$IC_C$$ from Eqs. 1, 2. For each plot, $$T = 0 $$ is in black and and $$T=3.64 \times 10^{-6} $$ is in red. The level of cell–cell contact is given for each column with (**A**, **E**, **I**,**M**, **Q**) $$C=0$$ cells, (**B**, **F**, **J**, **N**, **R**) $$C=1$$ cells, (**C**, **G**, **K**, **O**, **S**) $$C=2$$ cells, and (**D**, **H**, **L**, **P**, **T**) $$C=6$$ cells (color figure online)

**Fig. 15:**
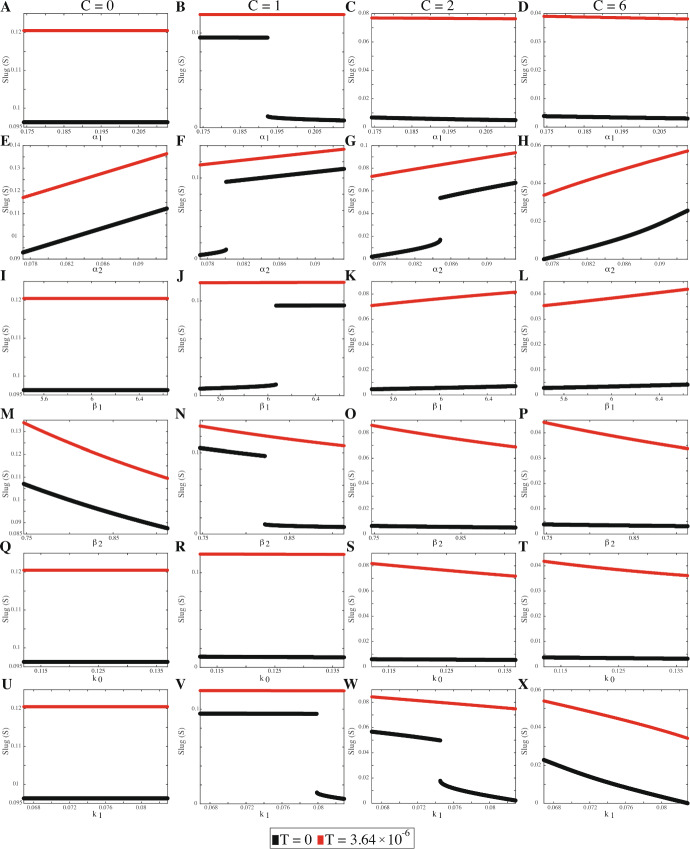
Slug (*S*) monotonicity plots for parameters (**A**–**D**) $$\alpha _1$$, (**E**–**H**) $$\alpha _2$$, (**I**–**L**) $$\beta _1$$, (**M**–**P**) $$\beta _2$$, (**Q**–**T**) $$k_0$$, and (**U**–**X**) $$k_1$$ from Eqs. 1, 2. For each plot, $$T=0 $$ is in black and and $$T=3.64 \times 10^{-6} $$ is in red. The level of cell–cell contact is given for each column with (**A**, **E**, **I**, **M**, **Q**, **U**) $$C=0$$ cells, (**B**, **F**, **J**, **N**, **R**, **V**) $$C=1$$ cells, (**C**, **G**, **K**, **O**, **S**, **W**) $$C=2$$ cells, and (**D**, **H**, **L**, **P**, **T**, **X**) $$C=6$$ cells (color figure online)

**Fig. 16:**
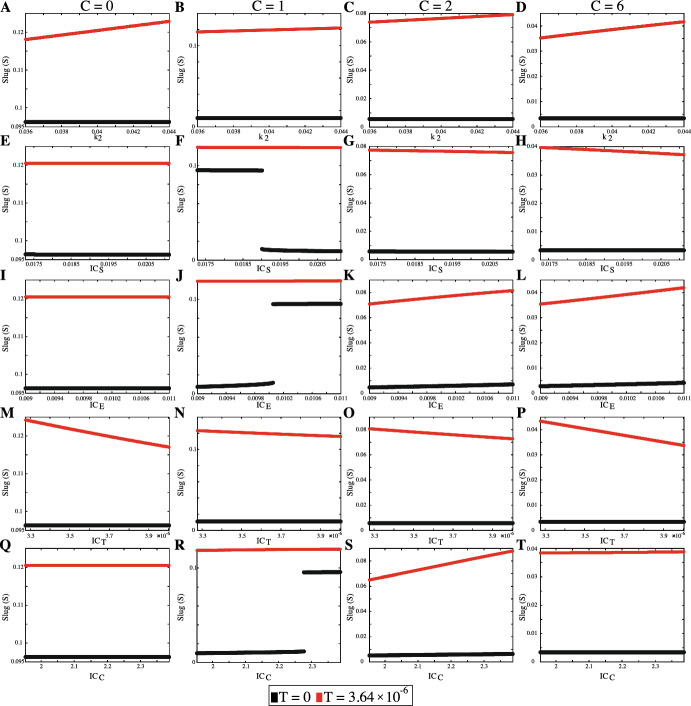
Slug (*S*) monotonicity plots for parameters (**A**–**D**) $$k_2$$, (**E**–**H**) $$IC_S$$, (**I**–**L**) $$IC_E$$, (**M**–**P**) $$IC_T$$, and (**Q**–**T**) $$IC_C$$ from Eqs. 1, 2. For each plot, $$T = 0 $$ is in black and and $$T=3.64 \times 10^{-6} $$ is in red. The level of cell–cell contact is given for each column with (**A**, **E**, **I**, **M**, **Q**) $$C=0$$ cells, (**B**, **F**, **J**, **N**, **R**) $$C=1$$ cells, (**C**, **G**, **K**, **O**, **S**) $$C=2$$ cells, and (**D**, **H**, **L**, **P**, **T**) $$C=6$$ cells (color figure online)

**Fig. 17:**
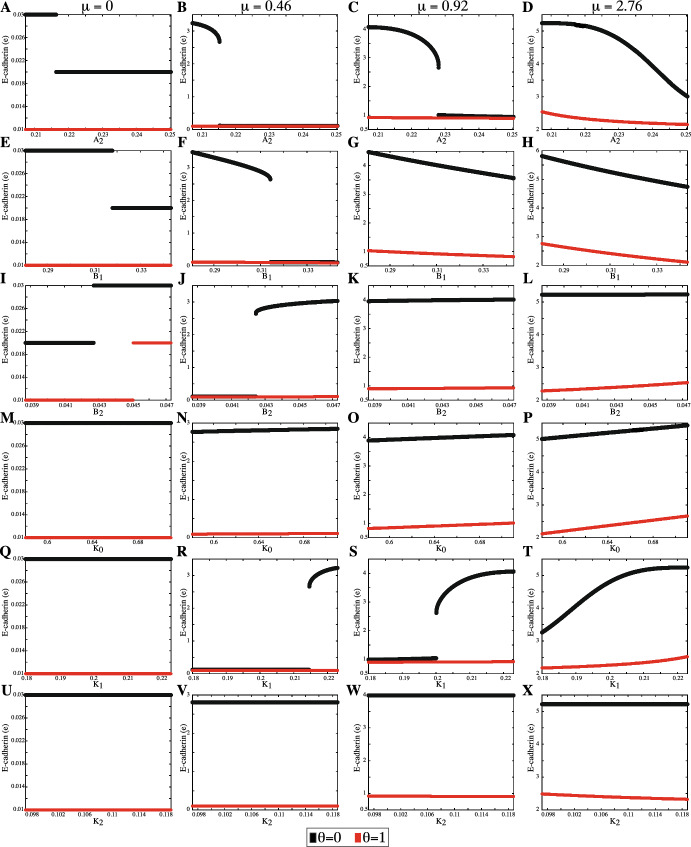
E-cadherin (*e*) monotonicity plots for parameters (**A**–**D**) $A_2$, (**E**–**H**) $B_1$, (**I**–**L**) $B_2$, (**M**–**P**) $K_0$, (**Q**–**T**) $K_1$, and (**U**–**X**) $K_2$ from nondimensionalized Set 1. For each plot, $\theta =0$ is in black and and $\theta = 1$ is in red. The level of cell–cell contact is given for each column with (**A**, **E**, **I**, **M**, **Q**, **U**) $\mu =0$, (**B**, **F**, **J**, **N**, **R**, **V**) $\mu =0.46$, (**C**, **G**, **K**, **O**, **S**, **W**) $\mu =0.92$, and (**D**, **H**, **L**, **P**, **T**, **X**) $\mu =2.76$ (color figure online)

**Fig. 18:**
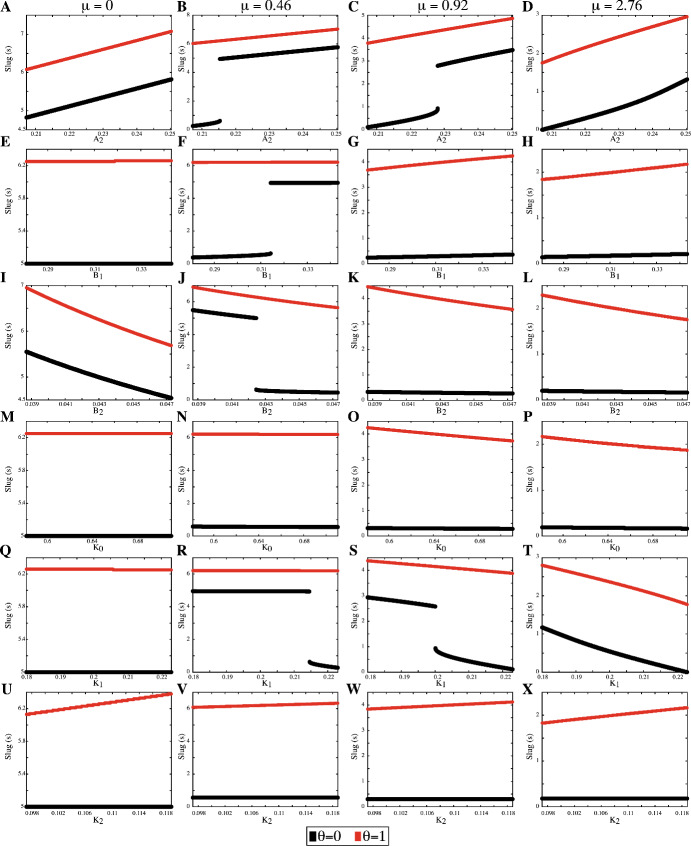
Slug (*s*) monotonicity plots for parameters (**A**–**D**) $A_2$, (**E**–**H**) $B_1$, (**I**–**L**) $B_2$, (**M**–**P**) $K_0$, (**Q**–**T**) $K_1$, and (**U**–**X**) $K_2$ from nondimensionalized Set 1. For each plot, $\theta =0$ is in black and and $\theta = 1$ is in red. The level of cell–cell contact is given for each column with (**A**, **E**, **I**, **M**, **Q**, **U**) $\mu =0$, (**B**, **F**, **J**, **N**, **R**, **V**) $\mu =0.46$, (**C**, **G**, **K**, **O**, **S**, **W**) $\mu =0.92$, and (**D**, **H**, **L**, **P**, **T**, **X**) $\mu =2.76$ (color figure online)

**Fig. 19:**
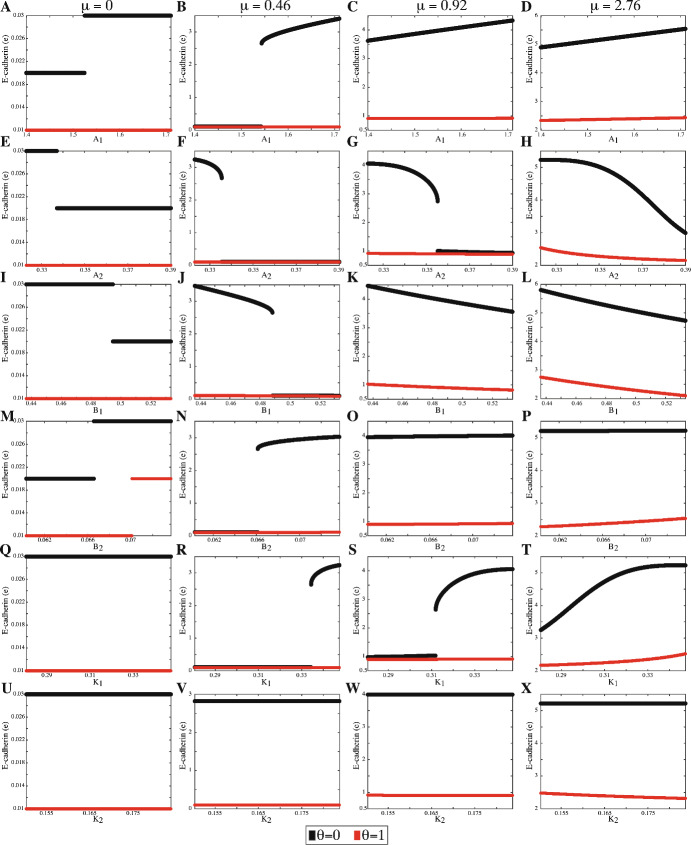
E-cadherin (*e*) monotonicity plots for parameters (**A**–**D**) $A_1$, (**E**–**H**) $A_2$, (**I**–**L**) $B_1$, (**M**–**P**) $B_2$, (**Q**–**T**) $K_1$, and (**U**–**X**) $K_2$ from nondimensionalized Set 2. For each plot, $\theta =0$ is in black and and $\theta = 1$ is in red. The level of cell–cell contact is given for each column with (**A**, **E**, **I**, **M**, **Q**, **U**) $\mu =0$, (**B**, **F**, **J**, **N**, **R**, **V**) $\mu =0.46$, (**C**, **G**, **K**, **O**, **S**, **W**) $\mu =0.92$, and (**D**, **H**, **L**, **P**, **T**, **X**) $\mu =2.76$ (color figure online)

**Fig. 20:**
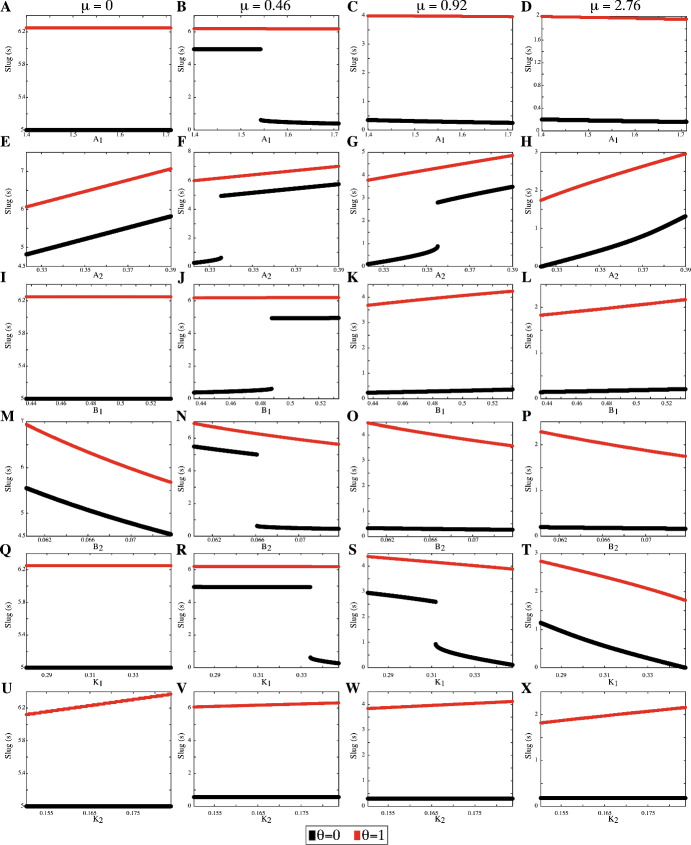
Slug (*s*) monotonicity plots for parameters (**A**–**D**) $A_1$, (**E**–**H**) $A_2$, (**I**–**L**) $B_1$, (**M**–**P**) $B_2$, (**Q**–**T**) $K_1$, and (**U**–**X**) $K_2$ from nondimensionalized Set 2. For each plot, $\theta =0$ is in black and and $\theta = 1$ is in red. The level of cell–cell contact is given for each column with (**A**, **E**, **I**, **M**, **Q**, **U**) $\mu =0$, (**B**, **F**, **J**, **N**, **R**, **V**) $\mu =0.46$, (**C**, **G**, **K**, **O**, **S**, **W**) $\mu =0.92$, and (**D**, **H**, **L**, **P**, **T**, **X**) $\mu =2.76$ (color figure online)

**Fig. 21:**
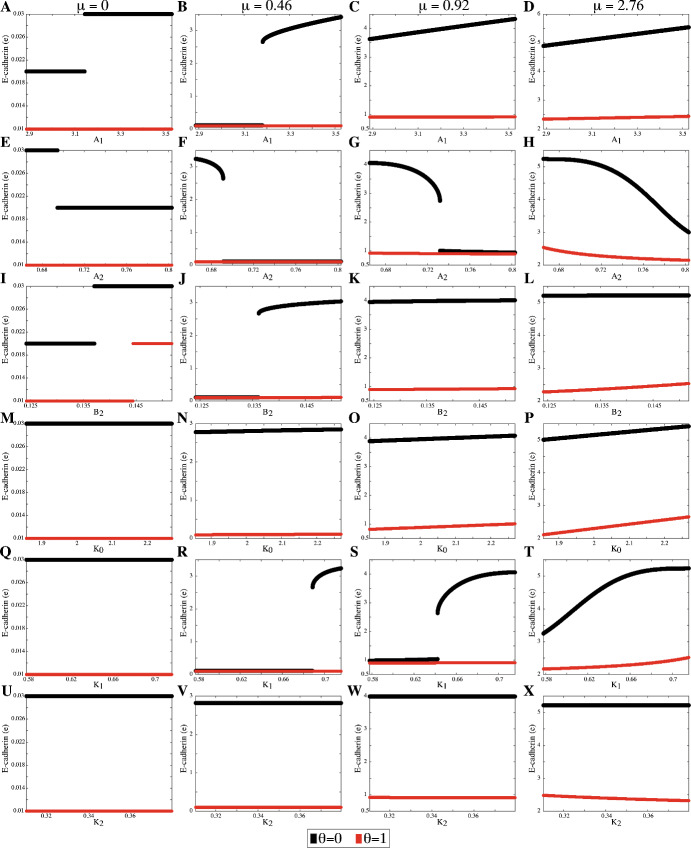
E-cadherin (*e*) monotonicity plots for parameters (**A**–**D**) $A_1$, (**E**–**H**) $A_2$, (**I**–**L**) $B_2$, (**M**–**P**) $K_0$, (**Q**–**T**) $K_1$, and (**U**–**X**) $K_2$ from nondimensionalized Set 3. For each plot, $\theta =0$ is in black and and $\theta = 1$ is in red. The level of cell–cell contact is given for each column with (**A**, **E**, **I**, **M**, **Q**, **U**) $\mu =0$, (**B**, **F**, **J**, **N**, **R**, **V**) $\mu =0.46$, (**C**, **G**, **K**, **O**, **S**, **W**) $\mu =0.92$, and (**D**, **H**, **L**, **P**, **T**, **X**) $\mu =2.76$ (color figure online)

**Fig. 22:**
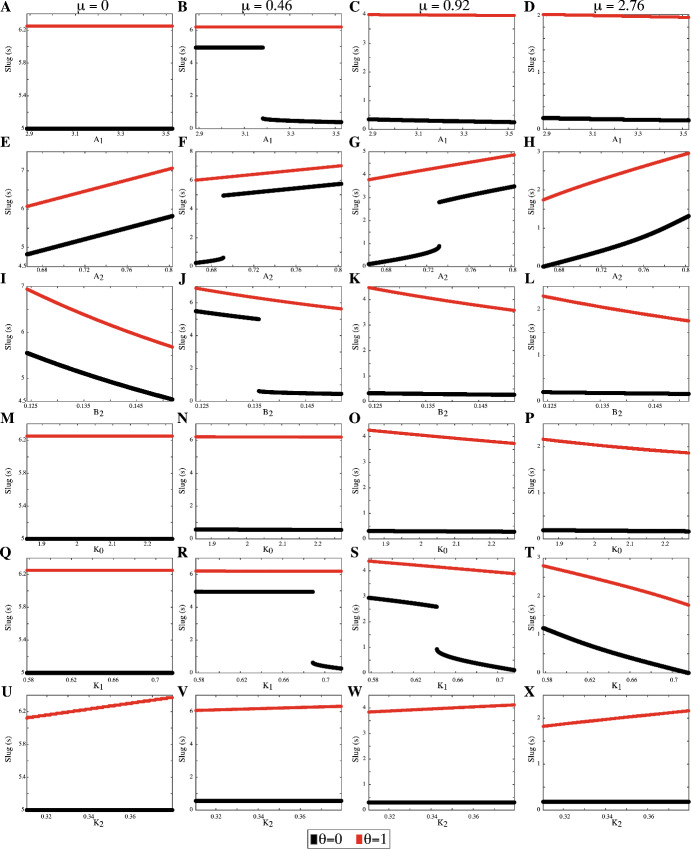
Slug (*s*) monotonicity plots for parameters (**A**–**D**) $A_1$, (**E**–**H**) $A_2$, (**I**–**L**) $B_2$, (**M**–**P**) $K_0$, (**Q**–**T**) $K_1$, and (**U**–**X**) $K_2$ from nondimensionalized Set 3. For each plot, $\theta =0$ is in black and and $\theta = 1$ is in red. The level of cell–cell contact is given for each column with (**A**, **E**, **I**, **M**, **Q**, **U**) $\mu =0$, (**B**, **F**, **J**, **N**, **R**, **V**) $\mu =0.46$, (**C**, **G**, **K**, **O**, **S**, **W**) $\mu =0.92$, and (**D**, **H**, **L**, **P**, **T**, **X**) $\mu =2.76$ (color figure online)

**Fig. 23:**
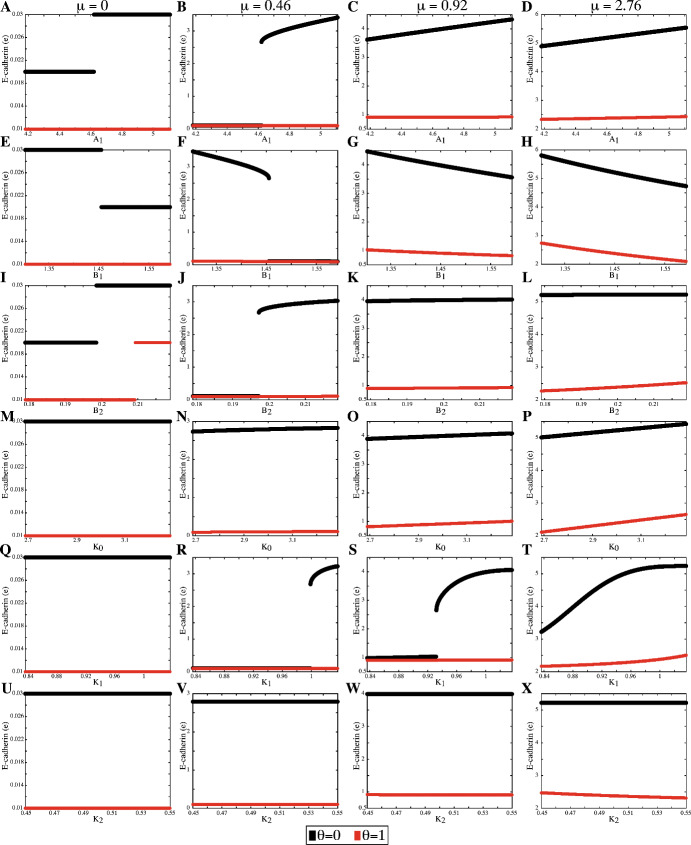
E-cadherin (*e*) monotonicity plots for parameters (**A**–**D**) $A_1$, (**E**–**H**) $B_1$, (**I**–**L**) $B_2$, (**M**–**P**) $K_0$, (**Q**–**T**) $K_1$, and (**U**–**X**) $K_2$ from nondimensionalized Set 4. For each plot, $\theta =0$ is in black and and $\theta = 1$ is in red. The level of cell–cell contact is given for each column with (**A**, **E**, **I**, **M**, **Q**, **U**) $\mu =0$, (**B**, **F**, **J**, **N**, **R**, **V**) $\mu =0.46$, (**C**, **G**, **K**, **O**, **S**, **W**) $\mu =0.92$, and (**D**, **H**, **L**, **P**, **T**, **X**) $\mu =2.76$ (color figure online)

**Fig. 24:**
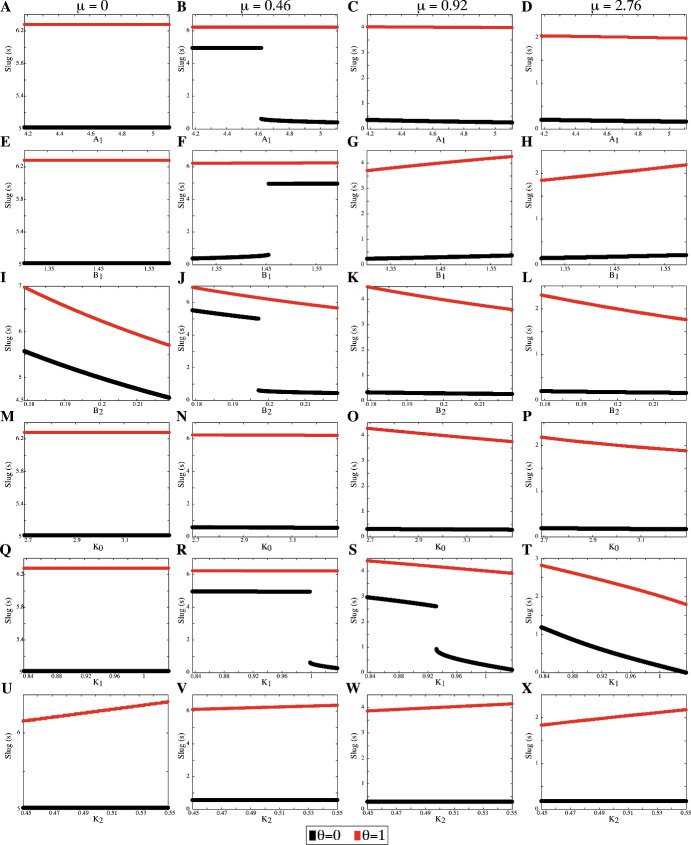
Slug (*s*) monotonicity plots for parameters (**A**–**D**) $A_1$, (**E**–**H**) $B_1$, (**I**–**L**) $B_2$, (**M**–**P**) $K_0$, (**Q**–**T**) $K_1$, and (**U**–**X**) $K_2$ from nondimensionalized Set 4. For each plot, $\theta =0$ is in black and and $\theta = 1$ is in red. The level of cell–cell contact is given for each column with (**A**, **E**, **I**, **M**, **Q**, **U**) $\mu =0$, (**B**, **F**, **J**, **N**, **R**, **V**) $\mu =0.46$, (**C**, **G**, **K**, **O**, **S**, **W**) $\mu =0.92$, and (**D**, **H**, **L**, **P**, **T**, **X**) $\mu =2.76$ (color figure online)

**Fig. 25:**
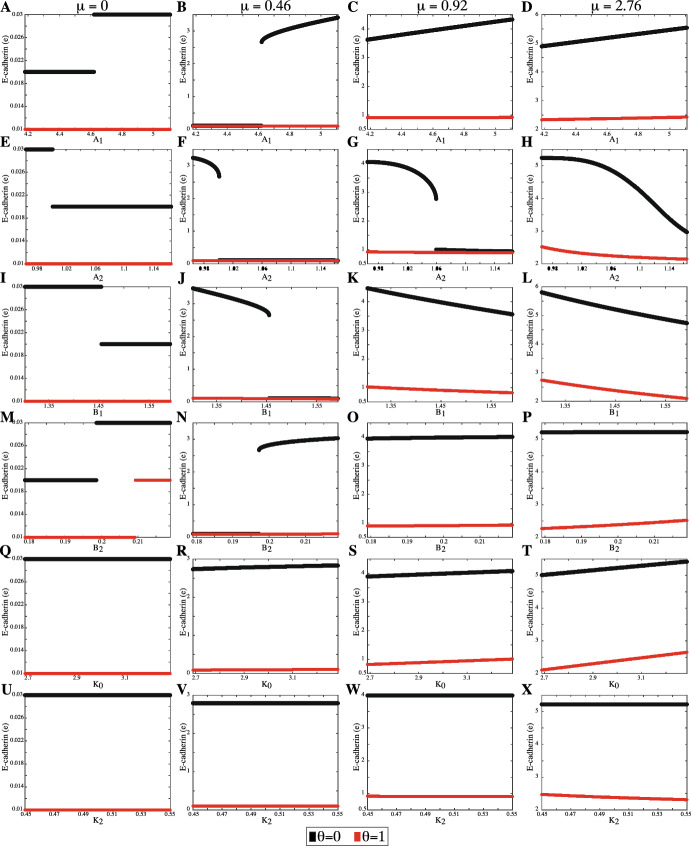
E-cadherin (*e*) monotonicity plots for parameters (**A**–**D**) $A_1$, (**E**–**H**) $A_2$, (**I**–**L**) $B_1$, (**M**–**P**) $B_2$, (**Q**–**T**) $K_0$, and (**U**–**X**) $K_2$ from nondimensionalized Set 5. For each plot, $\theta =0$ is in black and and $\theta = 1$ is in red. The level of cell–cell contact is given for each column with (**A**, **E**, **I**, **M**, **Q**, **U**) $\mu =0$, (**B**, **F**, **J**, **N**, **R**, **V**) $\mu =0.46$, (**C**, **G**, **K**, **O**, **S**, **W**) $\mu =0.92$, and (**D**, **H**, **L**, **P**, **T**, **X**) $\mu =2.76$ (color figure online)

**Fig. 26:**
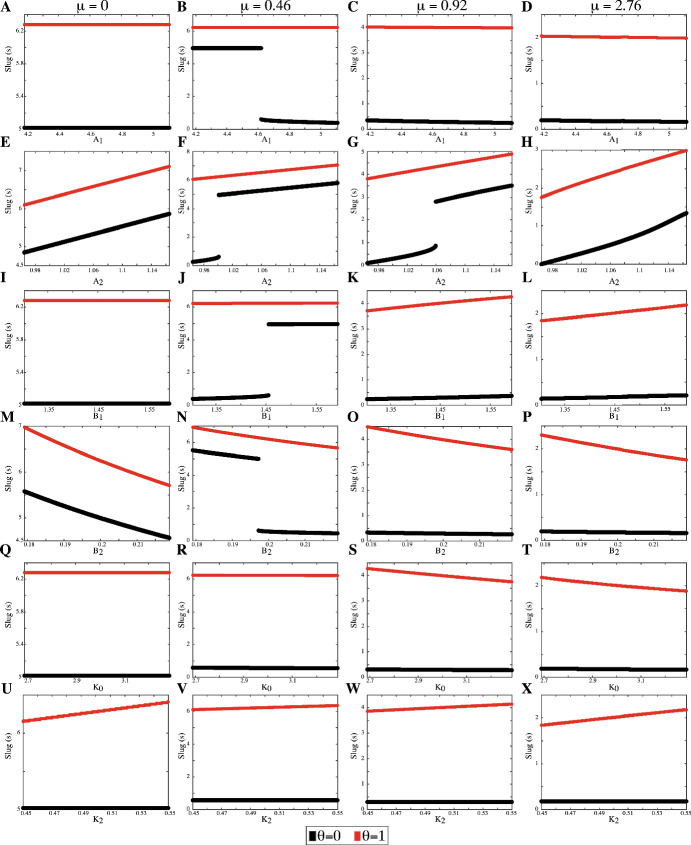
Slug (*s*) monotonicity plots for parameters (**A**–**D**) $A_1$, (**E**–**H**) $A_2$, (**I**–**L**) $B_1$, (**M**–**P**) $B_2$, (**Q**–**T**) $K_0$, and (**U**–**X**) $K_2$ from nondimensionalized Set 5. For each plot, $\theta =0$ is in black and and $\theta = 1$ is in red. The level of cell–cell contact is given for each column with (**A**, **E**, **I**, **M**, **Q**, **U**) $\mu =0$, (**B**, **F**, **J**, **N**, **R**, **V**) $\mu =0.46$, (**C**, **G**, **K**, **O**, **S**, **W**) $\mu =0.92$, and (**D**, **H**, **L**, **P**, **T**, **X**) $\mu =2.76$ (color figure online)

**Fig. 27:**
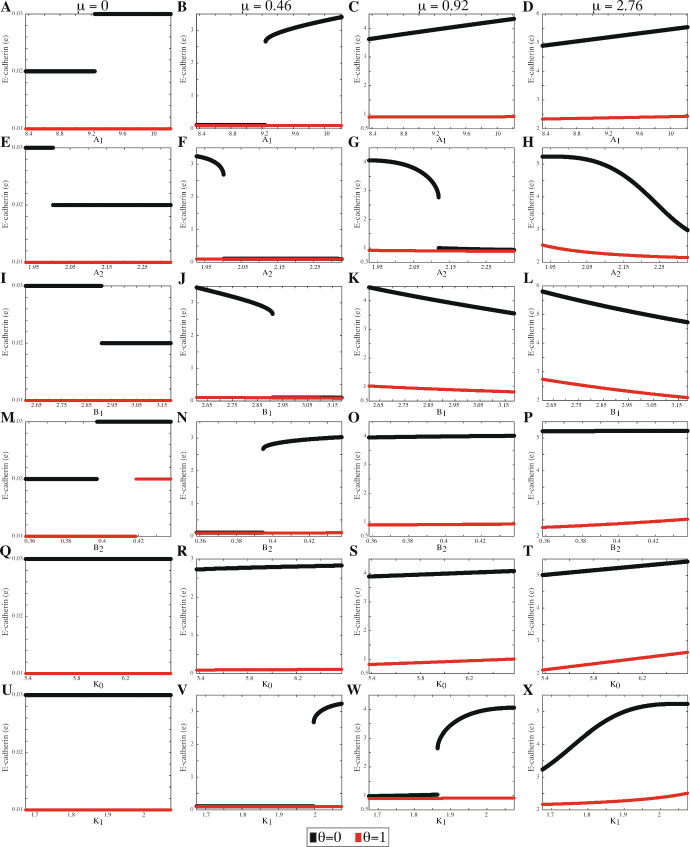
E-cadherin (*e*) monotonicity plots for parameters (**A**–**D**) $A_1$, (**E**–**H**) $A_2$, (**I**–**L**) $B_1$, (**M**–**P**) $B_2$, (**Q**–**T**) $K_0$, and (**U**–**X**) $K_1$ from nondimensionalized Set 6. For each plot, $\theta =0$ is in black and and $\theta = 1$ is in red. The level of cell–cell contact is given for each column with (**A**, **E**, **I**, **M**, **Q**, **U**) $\mu =0$, (**B**, **F**, **J**, **N**, **R**, **V**) $\mu =0.46$, (**C**, **G**, **K**, **O**, **S**, **W**) $\mu =0.92$, and (**D**, **H**, **L**, **P**, **T**, **X**) $\mu =2.76$ (color figure online)

**Fig. 28:**
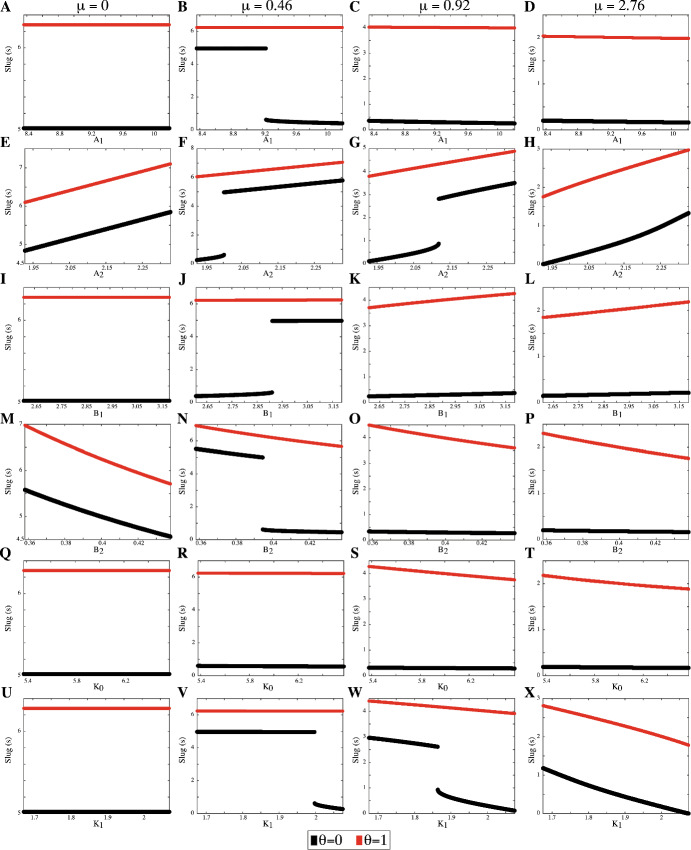
Slug (*s*) monotonicity plots for parameters (**A**–**D**) $A_1$, (**E**–**H**) $A_2$, (**I**–**L**) $B_1$, (**M**–**P**) $B_2$, (**Q**–**T**) $K_0$, and (**U**–**X**) $K_1$ from nondimensionalized Set 6. For each plot, $\theta =0$ is in black and and $\theta = 1$ is in red. The level of cell–cell contact is given for each column with (**A**, **E**, **I**, **M**, **Q**, **U**) $\mu =0$, (**B**, **F**, **J**, **N**, **R**, **V**) $\mu =0.46$, (**C**, **G**, **K**, **O**, **S**, **W**) $\mu =0.92$, and (**D**, **H**, **L**, **P**, **T**, **X**)$\mu =2.76$ (color figure online)

**Fig. 29:**
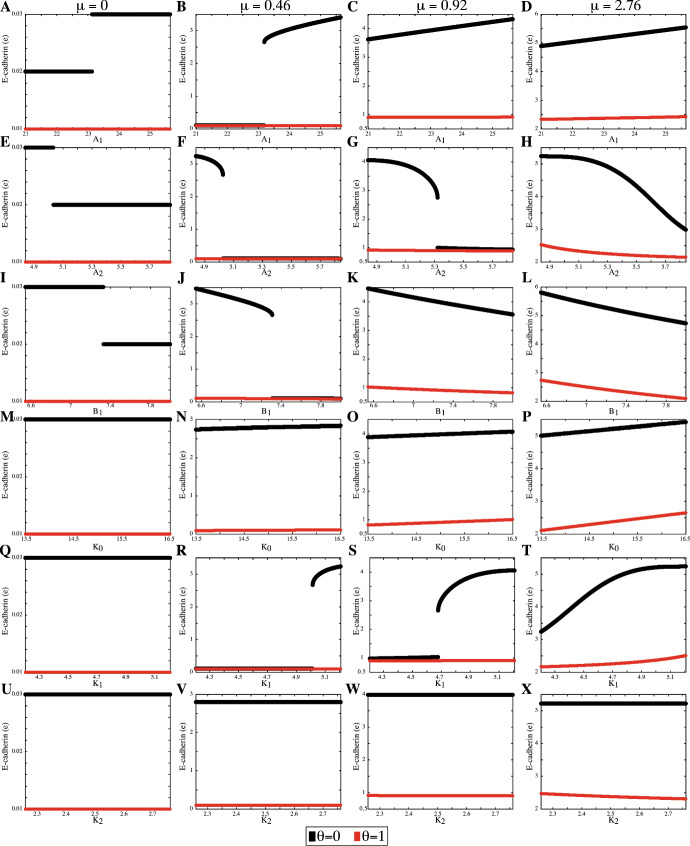
E-cadherin (*e*) monotonicity plots for parameters (**A**–**D**) $A_1$, (**E**–**H**) $A_2$, (**I**–**L**) $B_1$, (**M**–**P**) $K_0$, (**Q**–**T**) $K_1$, and (**U**–**X**) $K_2$ from nondimensionalized Set 7. For each plot, $\theta =0$ is in black and and $\theta = 1$ is in red. The level of cell–cell contact is given for each column with (**A**, **E**, **I**, **M**, **Q**, **U**) $\mu =0$, (**B**, **F**, **J**, **N**, **R**, **V**) $\mu =0.46$, (**C**, **G**, **K**, **O**, **S**, **W**) $\mu =0.92$, and (**D**, **H**, **L**, **P**, **T**, **X**) $\mu =2.76$ (color figure online)

**Fig. 30:**
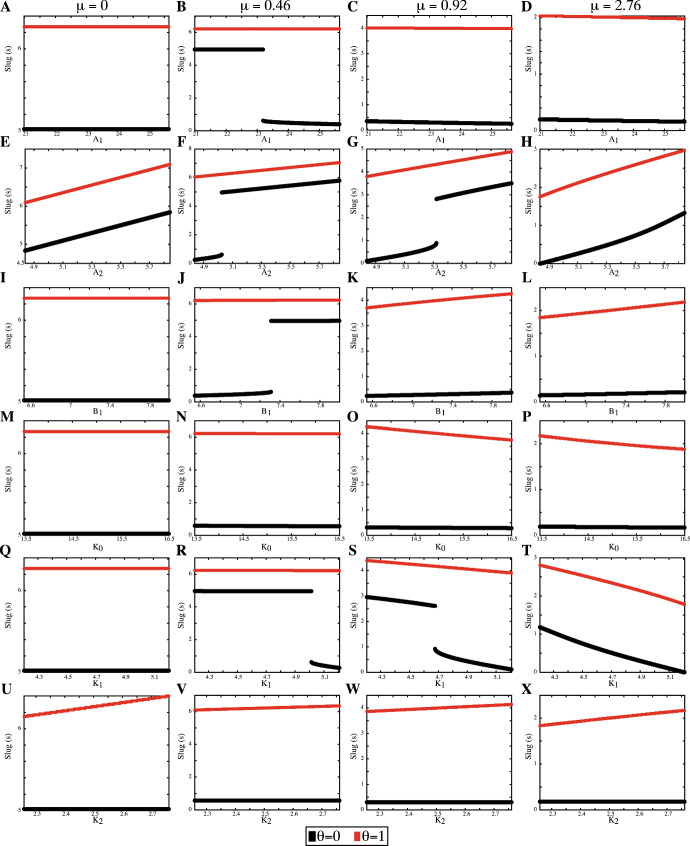
Slug (*s*) monotonicity plots for parameters (**A**–**D**) $A_1$, (**E**–**H**) $A_2$, (**I**–**L**) $B_1$, (**M**–**P**) $K_0$, (**Q**–**T**) $K_1$, and (**U**–**X**) $K_2$ from nondimensionalized Set 7. For each plot, $\theta =0$ is in black and and $\theta = 1$ is in red. The level of cell–cell contact is given for each column with (**A**, **E**, **I**, **M**, **Q**, **U**) $\mu =0$, (**B**, **F**, **J**, **N**, **R**, **V**) $\mu =0.46$, (**C**, **G**, **K**, **O**, **S**, **W**) $\mu =0.92$, and (**D**, **H**, **L**, **P**, **T**, **X**) $\mu =2.76$ (color figure online)

**Fig. 31:**
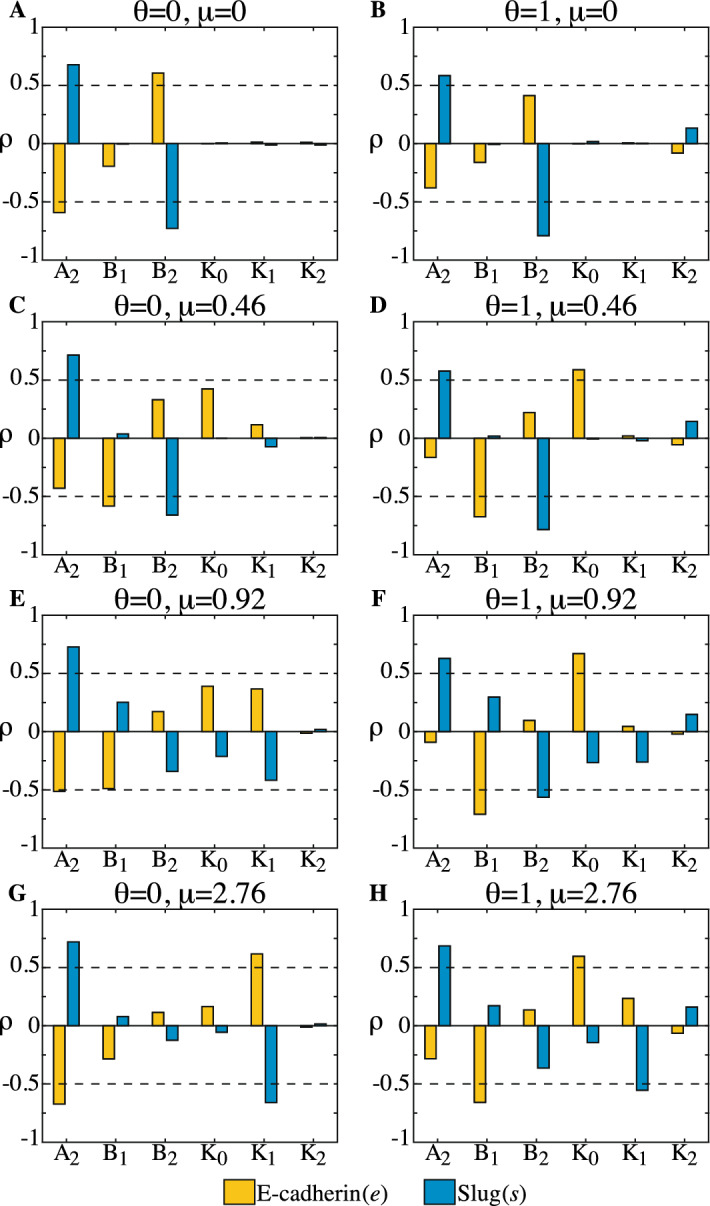
PRCC results for the steady state values of E-cadherin (*e*, gold) and Slug (*s*, blue) for nondimensionalized Set 1 under treatment groups (**A**) $(\theta ,\mu )=(0,0)$, (**B**) $(\theta ,\mu )=(1,0)$, (**C**) $(\theta ,\mu )=(0,0.46)$, (**D**) $(\theta ,\mu )=(1,0.46)$, (**E**) $(\theta ,\mu )=(0,0.92)$, (**F**) $(\theta ,\mu )=(1,0.92)$, (**G**) $(\theta ,\mu )=(0,2.76)$, and (**H**) $(\theta ,\mu )=(1, 2.76)$. A value of $|\rho |=0.5$ was used as a cut-off for significance, as marked by the dashed black lines in each figure (color figure online)

**Fig. 32:**
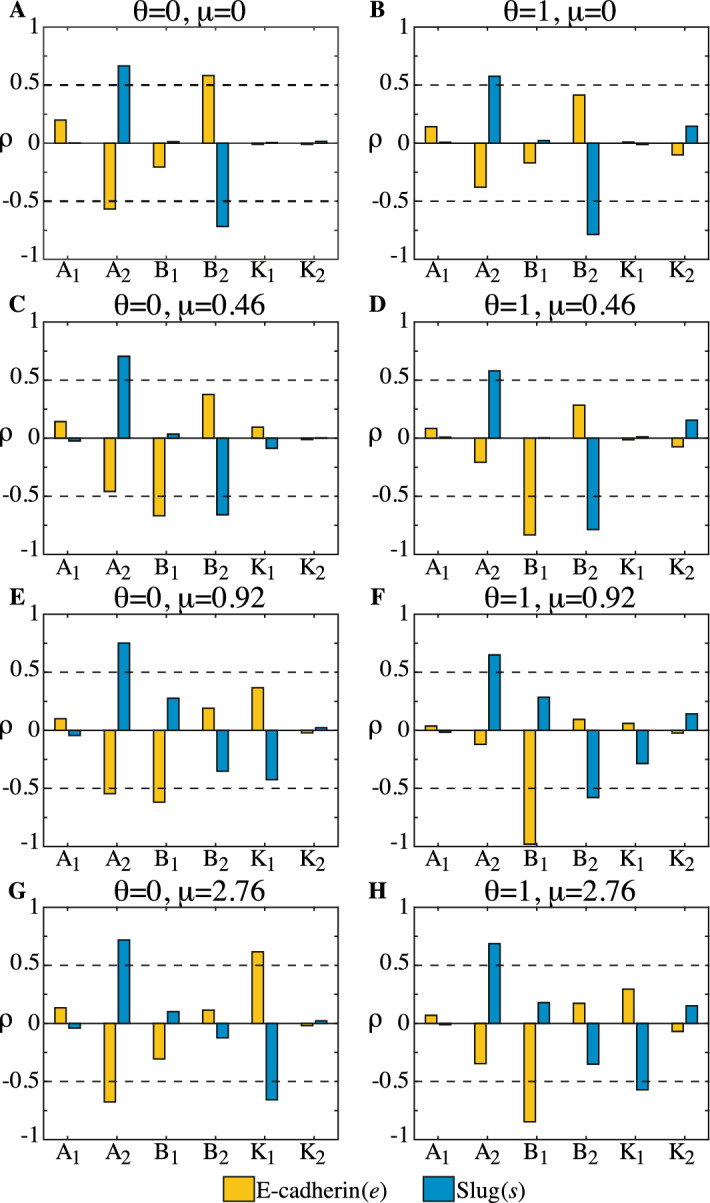
PRCC results for the steady state values of E-cadherin (*e*, gold) and Slug (*s*, blue) for nondimensionalized Set 2 under treatment groups (**A**) $(\theta ,\mu )=(0,0)$, (**B**) $(\theta ,\mu )=(1,0)$, (**C**) $(\theta ,\mu )=(0,0.46)$, (**D**) $(\theta ,\mu )=(1,0.46)$, (**E**) $(\theta ,\mu )=(0,0.92)$, (**F**) $(\theta ,\mu )=(1,0.92)$, (**G**) $(\theta ,\mu )=(0,2.76)$, and (**H**) $(\theta ,\mu )=(1, 2.76)$. A value of $|\rho |=0.5$ was used as a cut-off for significance, as marked by the dashed black lines in each figure (color figure online)

**Fig. 33:**
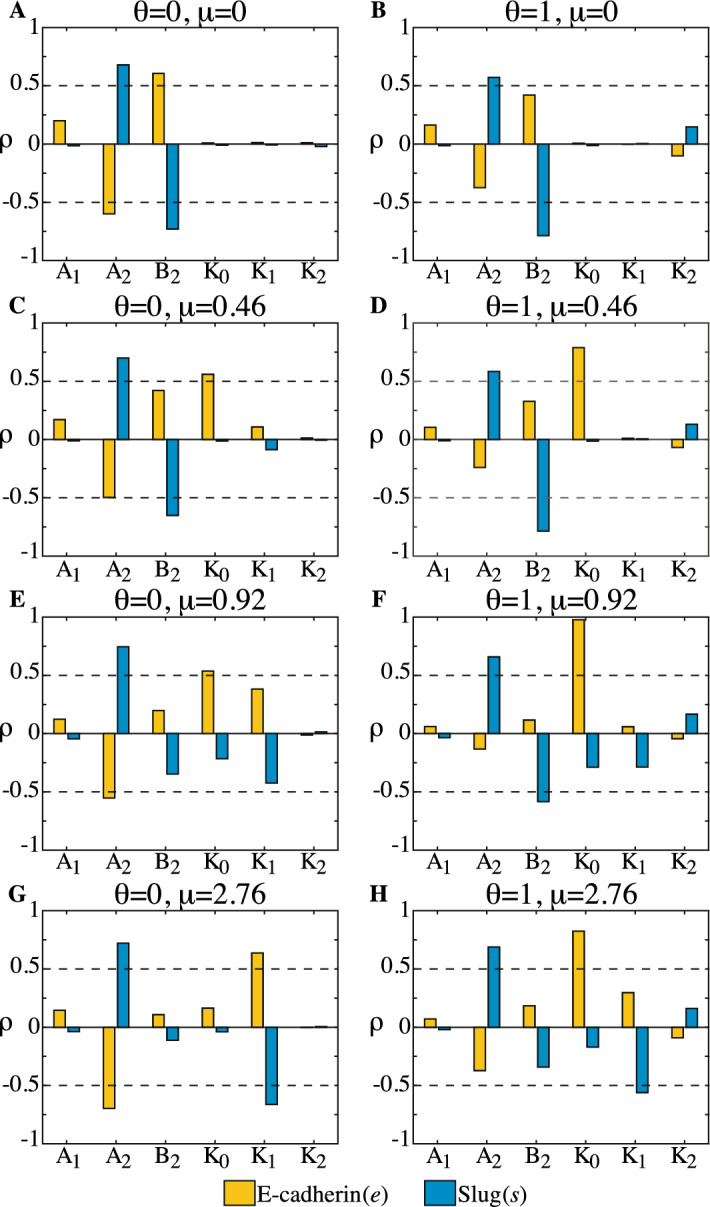
PRCC results for the steady state values of E-cadherin (*e*, gold) and Slug (*s*, blue) for nondimensionalized Set 3 under treatment groups (**A**) $(\theta ,\mu )=(0,0)$, (**B**) $(\theta ,\mu )=(1,0)$, (**C**) $(\theta ,\mu )=(0,0.46)$, (**D**) $(\theta ,\mu )=(1,0.46)$, (**E**) $(\theta ,\mu )=(0,0.92)$, (**F**) $(\theta ,\mu )=(1,0.92)$, (**G**) $(\theta ,\mu )=(0,2.76)$, and (**H**) $(\theta ,\mu )=(1, 2.76)$. A value of $|\rho |=0.5$ was used as a cut-off for significance, as marked by the dashed black lines in each figure (color figure online)

**Fig. 34:**
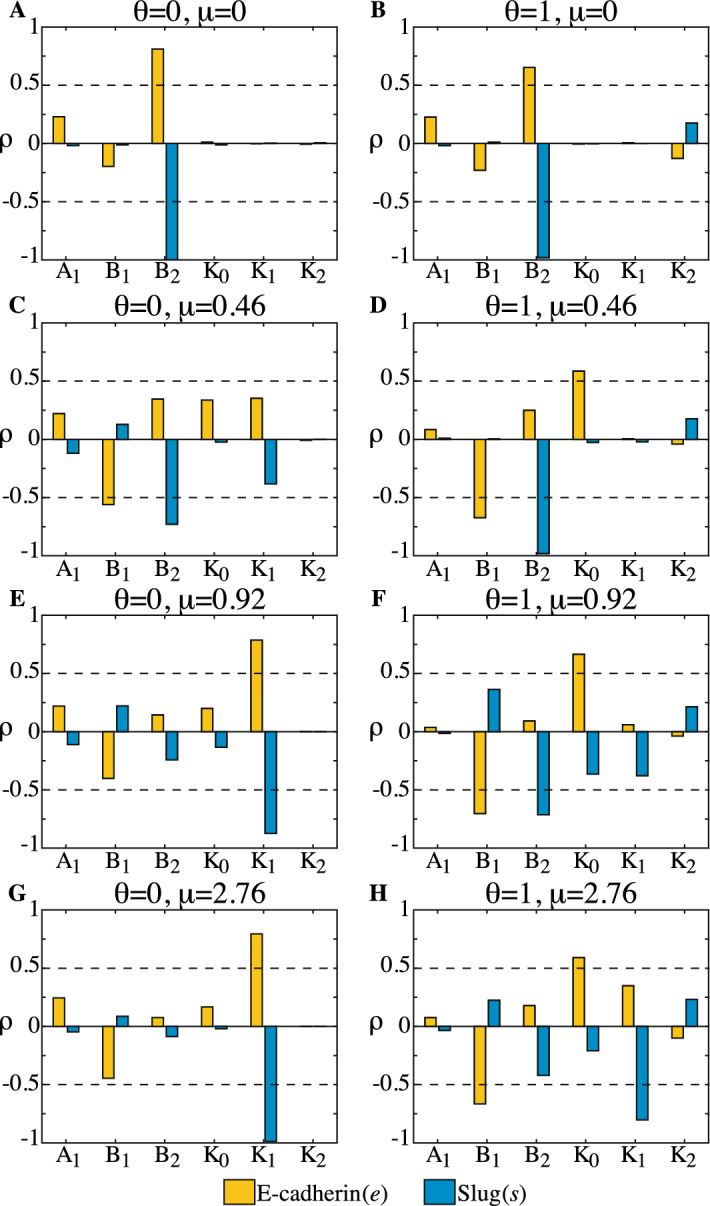
PRCC results for the steady state values of E-cadherin (*e*, gold) and Slug (*s*, blue) for nondimensionalized Set 4 under treatment groups (**A**) $(\theta ,\mu )=(0,0)$, (**B**) $(\theta ,\mu )=(1,0)$, (**C**) $(\theta ,\mu )=(0,0.46)$, (**D**) $(\theta ,\mu )=(1,0.46)$, (**E**) $(\theta ,\mu )=(0,0.92)$, (**F**) $(\theta ,\mu )=(1,0.92)$, (**G**) $(\theta ,\mu )=(0,2.76)$, and (**H**) $(\theta ,\mu )=(1, 2.76)$. A value of $|\rho |=0.5$ was used as a cut-off for significance, as marked by the dashed black lines in each figure (color figure online)

**Fig. 35:**
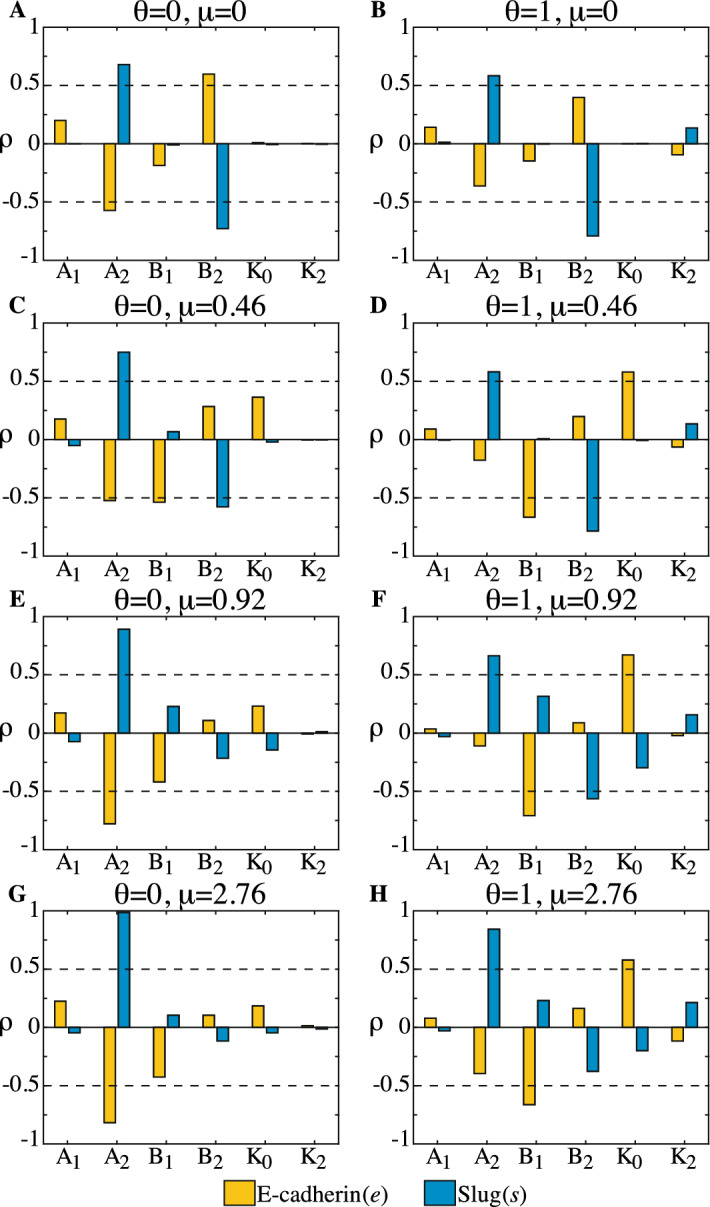
PRCC results for the steady state values of E-cadherin (*e*, gold) and Slug (*s*, blue) for nondimensionalized Set 5 under treatment groups (**A**) $(\theta ,\mu )=(0,0)$, (**B**) $(\theta ,\mu )=(1,0)$, (**C**) $(\theta ,\mu )=(0,0.46)$, (**D**) $(\theta ,\mu )=(1,0.46)$, (**E**) $(\theta ,\mu )=(0,0.92)$, (**F**) $(\theta ,\mu )=(1,0.92)$, (**G**) $(\theta ,\mu )=(0,2.76)$, and (**H**) $(\theta ,\mu )=(1, 2.76)$. A value of $|\rho |=0.5$ was used as a cut-off for significance, as marked by the dashed black lines in each figure (color figure online)

**Fig. 36:**
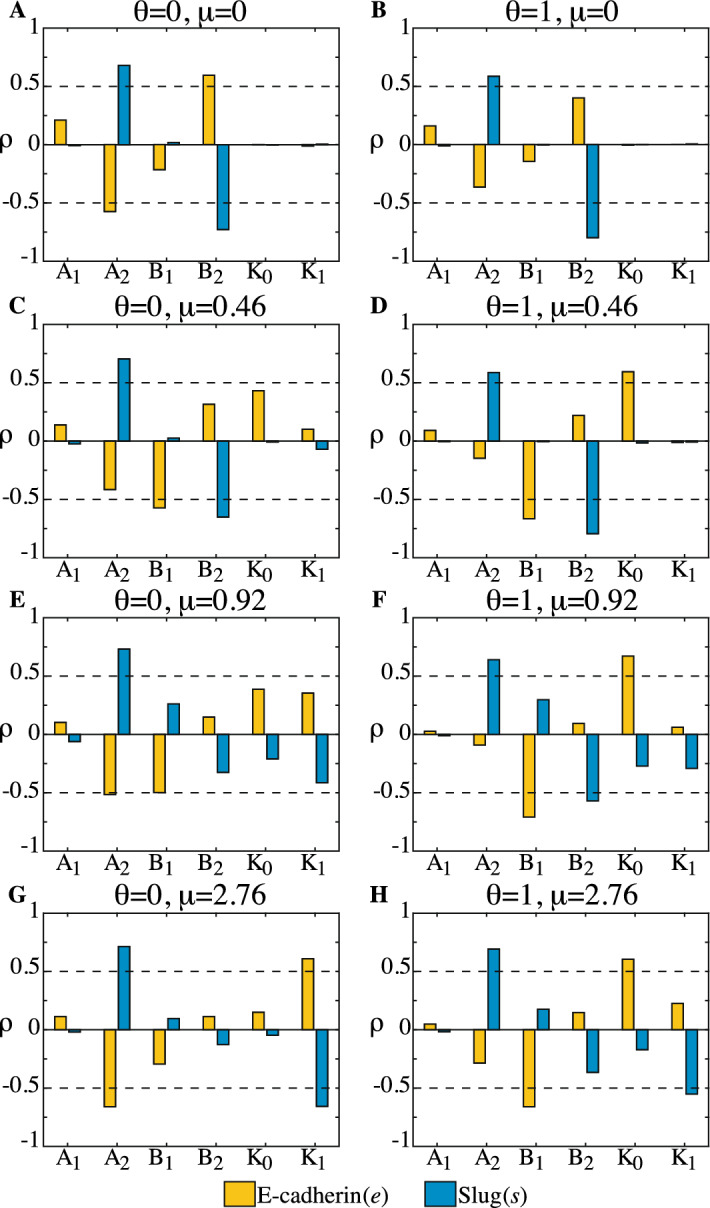
PRCC results for the steady state values of E-cadherin (*e*, gold) and Slug (*s*, blue) for nondimensionalized Set 6 under treatment groups (**A**) $(\theta ,\mu )=(0,0)$, (**B**) $(\theta ,\mu )=(1,0)$, (**C**) $(\theta ,\mu )=(0,0.46)$, (**D**) $(\theta ,\mu )=(1,0.46)$, (**E**) $(\theta ,\mu )=(0,0.92)$, (**F**) $(\theta ,\mu )=(1,0.92)$, (**G**) $(\theta ,\mu )=(0,2.76)$, and (**H**) $(\theta ,\mu )=(1, 2.76)$. A value of $|\rho |=0.5$ was used as a cut-off for significance, as marked by the dashed black lines in each figure (color figure online)

**Fig. 37:**
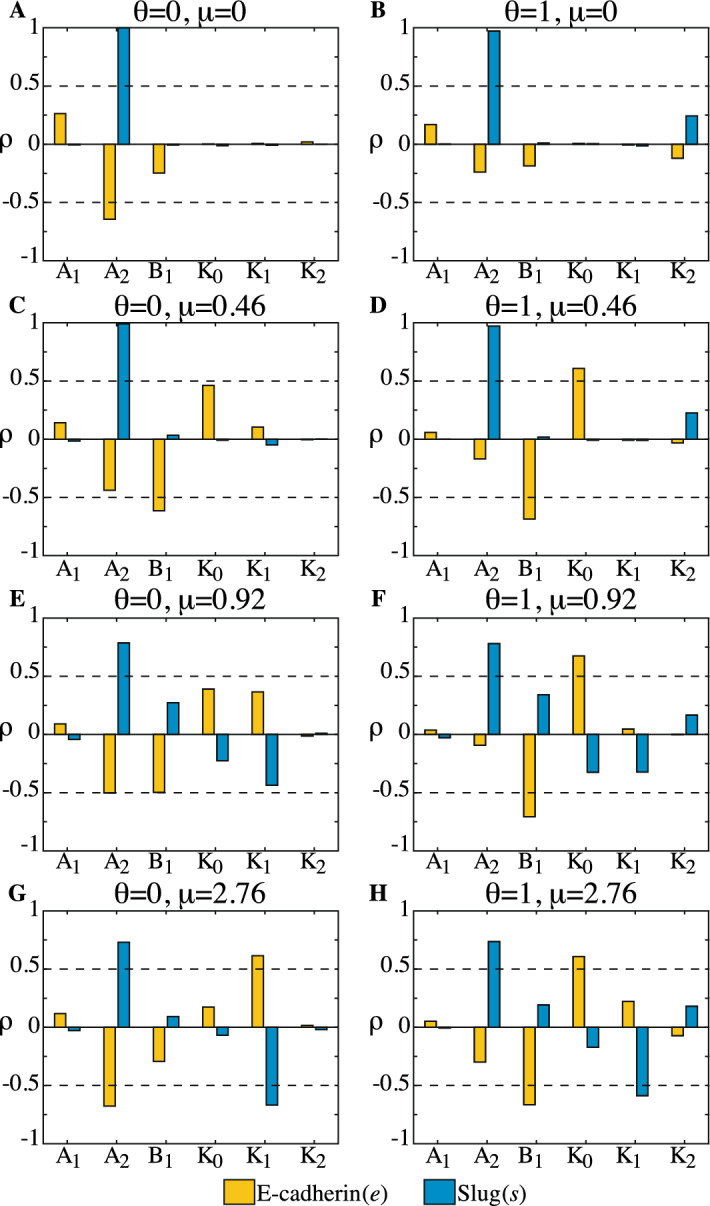
PRCC results for the steady state values of E-cadherin (*e*, gold) and Slug (*s*, blue) for nondimensionalized Set 7 under treatment groups (**A**) $(\theta ,\mu )=(0,0)$, (**B**) $(\theta ,\mu )=(1,0)$, (**C**) $(\theta ,\mu )=(0,0.46)$, (**D**) $(\theta ,\mu )=(1,0.46)$, (**E**) $(\theta ,\mu )=(0,0.92)$, (**F**) $(\theta ,\mu )=(1,0.92)$, (**G**) $(\theta ,\mu )=(0,2.76)$, and (**H**) $(\theta ,\mu )=(1, 2.76)$. A value of $|\rho |=0.5$ was used as a cut-off for significance, as marked by the dashed black lines in each figure (color figure online)

## Appendix

Figures 6, 7, 8, 9, 10, 11: Bifurcation plots for E-cadherin (*e*) and Slug (*s*) with respect to $\theta$ at varying levels of cell–cell contact ($\mu$) for nondimensionalized Sets 1–7. (A&B) Cells without cellular contact ($\mu =0$) begin in the mesenchymal state with low membrane E-cadherin and a high Slug. Exogenous TGF-$\beta$ ($\theta$) lowers *e* and increases *s* but does not produce a switch. (C-F) Cells with 1, 2 neighbors ($\mu =0.46$, 0.92) begin in the epithelial state. Increasing $\theta$ reduces E-cadherin and increases Slug and, eventually, the cell will undergo the irreversible transition to the mesenchymal steady state. (G&H) Epithelial cells with 6 neighbors ($\mu =2.76$) are unable to transition as $\theta$ is increased.

Figure 12: Bifurcation plots for E-cadherin (*e*) and Slug (*s*) with respect to $\mu$ with $\theta =0$ for nondimensionalized Sets 1–7. Cells with $\mu =2.76$ begin in the epithelial steady state. Loss of cellular contact results in a bistable switch to the mesenchymal steady state. If the cell is able to regain cellular neighbors, it could ultimately switch back to the epithelial steady state.

Figures 13, 14, 15, 16: Monotonicity plots for the steady state value of E-cadherin (*E*) and Slug (*S*) with respect to parameters from the model in Eqs. 1, 2. E-cadherin monotonicity plots are presented in Figs. 13, 14 and the Slug monotonicity plots are presented in A10–11. Parameter ranges are listed in Table 1. For each figure, the cell–cell contact is varied by column ($$C=0$$, 1, 2, and 6 cells) while each individual subfigure has $$T=0 $$ in black and $$T=3.64 \times 10^{-6} $$ in red.

Figures 17, 18, 19, 20, 21, 22, 23, 24, 25, 26, 27, 28, 29, 30: Monotonicity Plots for the steady state value of E-cadherin (*e*) and Slug (*s*) for nondimensionalized Sets 1–7. Parameter ranges explored for each set are listed in Table 4. For each figure, the cell–cell contact is varied by column ($\mu =0$, 0.46, 0.92, and 2.76) while each individual subfigure has $\theta =0$ in black and $\theta =1$ in red.

Figures 31, 32, 33, 34, 35, 36, 37: PRCC results for the steady state values of E-cadherin (*e*) and Slug (*s*) for all nondimensionalized systems for four levels of cell–cell contact ($\mu =0$, 0.46, 0.92, 2.76) and two levels of TGF-$\beta$ ($\theta =0$, 1). A cut-off for parameter significance was $|\rho |=0.5$, as indicated by the dashed black lines in each subfigure. The results of these figures are summarized in Fig. 5.
